# Application of Lung-Targeted Lipid Nanoparticle-delivered mRNA of soluble PD-L1 via SORT Technology in Acute Respiratory Distress Syndrome

**DOI:** 10.7150/thno.86466

**Published:** 2023-09-04

**Authors:** Han Sun, Yu Zhang, Jiahui Wang, Juncheng Su, Dejian Zhou, Xiang Yu, Yingjie Xu, Wen Yang

**Affiliations:** 1 Department of Biochemistry and Molecular Cell Biology, Shanghai Key Laboratory for Tumor Microenvironment and Inflammation, Shanghai Jiao Tong University School of Medicine, Shanghai, 200025, China.; 2 Department of Gastrointestinal Surgery, Renji Hospital Affiliated, Shanghai Jiao Tong University School of Medicine, Shanghai, 200127, China.; 3 State Key Laboratory of Genetic Engineering, School of Life Sciences, Fudan University, Shanghai, 200433, China.; 4 Key Laboratory of Cell Differentiation and Apoptosis of Chinese Ministry of Education, Shanghai Jiao Tong University School of Medicine, Shanghai, 200025, China.; 5 Shanghai RNACure Biopharma Co., Ltd. Shanghai, 200438, China.; 6 Shanghai Frontiers Science Center of Cellular Homeostasis and Human Diseases. Shanghai, 200025, China.

**Keywords:** immunosuppression, PD-L1, mRNA therapy, LNPs, ARDS

## Abstract

**Rationale**: Acute respiratory distress syndrome (ARDS) is a life-threatening condition characterized by excessive immune response usually due to lung inflammation. Local immunosuppression is crucial for effective ARDS treatment. However, current methods are limited in their ability to target the lungs specifically.

**Methods**: This study utilized lung-targeted lipid nanoparticles (LNPs) with 1,2-dioleoyl-3-trimethylammonium-propane (termed DOTAP-LNPs) to encapsulate chemically modified soluble programmed death ligand-1 (sPD-L1) mRNA and examined its physiological characteristics and therapeutic efficacy. A comparative analysis was performed between sPD-L1 mRNA delivered by DOTAP-LNPs, sPD-L1 mRNA delivered by regular LNPs (MC3-LNPs), and PD-L1-Fc recombinant protein administered systemically. Additionally, the survival rate of ARDS mice treated with different drugs was assessed.

**Results:** Administration of sPD-L1 mRNA-LNPs to ARDS model mice significantly reduced leukocyte chemotaxis and protein accumulation in lung tissue, along with a decrease in pulmonary edema. Notably, *in situ* intervention using sPD-L1 mRNA-DOTAP-LNPs exhibited superior therapeutic effects compared to PD-L1-Fc recombinant protein and sPD-L1 mRNA encapsulated in MC3-LNPs. Importantly, treatment with sPD-L1 mRNA-DOTAP-LNPs improved the survival rate of ARDS model mice.

**Conclusion:** This study demonstrates the feasibility of utilizing stable and reliable mRNA to express the immunosuppressive molecule sPD-L1 specifically in the lungs. The findings provide proof of concept for localized nanoparticle delivery and offer a novel therapeutic strategy for treating acute inflammation in ARDS.

## Introduction

Acute respiratory distress syndrome (ARDS) is a severe respiratory disorder characterized by the presence of non-cardiogenic pulmonary edema, bilateral chest radiographic opacities, and severe hypoxemia with the etiology including pulmonary and extrapulmonary factors such as bacterial and viral pneumonia, aspiration, lung contusion, and severe systemic infections 1, 2. ARDS has a mortality rate exceeding 30% and affects about 10% of intensive care unit patients, particularly during the COVID-19 pandemic 3. Traditional treatments for ARDS, such as lung-protective ventilation and fluid-restrictive resuscitation, mainly provide artificial support without addressing the underlying lung injury 4, 5. These methods often involve invasive procedures and reliance on medical devices. Therefore, there is a need for novel anti-inflammatory strategies with high specificity and reduced off-target effects to combat ARDS effectively.

Recent studies have highlighted the crucial role of chemotaxis and activation of pro-inflammatory immune cells in the development of ARDS-associated lung injury 6. Programmed death ligand-1 (PD-L1) protein has shown significant potential in treating immune-related diseases. It functions by interacting with the PD-1 receptor on activated T cells, thereby suppressing T cell activation signals and promoting immune evasion 7-10. While the PD-L1-PD-1 axis has been extensively targeted in human cancer therapies with numerous clinical trials, its original role in maintaining immune homeostasis and restraining excessive immune responses remains underutilized. Previous research has revealed that PD-1-deficient mice are susceptible to lupus-like autoimmune diseases and autoimmune myocarditis 11. Recombinant fusion proteins of PD-L1 and adenoviruses expressing PD-L1 have shown reduced inflammation severity in various animal models, including rheumatoid arthritis, colitis, psoriasis, and ARDS 11-15, underscoring the involvement of the PD-1-PD-L1 axis in other immune regulatory capacities 16, 17. The soluble form of PD-L1 (sPD-L1) lacks the intracellular and transmembrane domains of PD-L1 and can bind to the PD-1 receptor independently of cell-to-cell contact, expanding its range of action. It has been reported that sPD-L1 is associated with the mortality of patients with direct ARDS and exhibits a protective effect in mice with acute lung injury 18, 19. Our previous research has further demonstrated that sPD-L1 induces the apoptosis of monocyte-derived macrophages (MDMs), contributing to its protective effects in ARDS 19. Therefore, we have selected sPD-L1 protein as the immunomodulatory effector for our study.

Protein therapy is accompanied by limitations, such as high cost, short half-life, and the potential for antibody formation against exogenous proteins. However, *in vitro* transcribed (IVT) mRNA therapy has emerged as a promising protein replacement approach, garnering significant attention. This innovative method incorporates crucial structural elements like the 5' cap, poly(A) tail, and especially various chemical modifications, endowing it with remarkable advantages, including rapid preparation, low immunogenicity, and high expression efficiency 20-25. IVT mRNA therapy has demonstrated impressive advancements in various fields, including viral vaccine development, cancer treatment, and genome editing, highlighting its broad potential in revolutionizing therapeutic interventions 26-28.

Due to the large molecular weight, negative charge, instability, and susceptibility to degradation by nucleases, mRNA molecules pose challenges for therapeutic applications. The use of lipid nanoparticles (LNPs) delivery platforms ensures stable *in vivo* delivery of mRNA and facilitates well expression. While achieving mRNA delivery, selective organ targeting (SORT) delivery platform implement specific delivery to lung tissue 29, 30. This tissue-specific delivery is accomplished through modifications of LNP formulations, which involve the binding of certain proteins in the blood, enabling recognition by cell receptors in specific organs and facilitating endocytosis. For example, apolipoprotein E (apoE) binds to the surface of MC3-LNPs, leading to their recognition by LDL-R receptors on liver cells and mediating the uptake of mRNA-MC3-LNPs by liver cells 31. Additionally, for DOTAP-LNPs, vitronectin has been reported to act as an endogenous targeting ligand for lung tissue, promoting intracellular mRNA delivery 32. The SORT delivery technology allows precise targeting of the affected area where the disease occurs, resulting in more effective disease control and minimizing the side effects associated with systemic protein expression. This breakthrough unlocks a diverse range of pharmaceutical applications for intravenous mRNA therapy.

In this study, we established a potent platform for tailored inflammation control via SORT LNP-based lung-targeted delivery of chemically modified sPD-L1 mRNA, which could be a promising strategy for ARDS treatment.

## Materials and Methods

### Cell culture

The human embryonic kidney cell line (HEK293T) and mouse hepatic cell line (AML12) were utilized to assess the expression level of sPD-L1. The A549 human lung cancer cell line is utilized for investigating the co-localization of lysosomes and mRNA-LNPs uptake. All cells were obtained from the American Type Culture Collection (ATCC). AML12 cells were cultured in Dulbecco's modified eagle medium/nutrient mixture F-12 (DMEM/F-12, L310KJ, BasalMedia), supplemented with 0.45% insulin-transferrin-selenium (ITS, S450J7, BasalMedia) and 10% fetal bovine serum (FBS, 10099141, Gibco). HEK293T cells were maintained in high-glucose DMEM (L110KJ, BasalMedia) and 10% FBS. A549 cells were cultured in RPMI 1640 (L210KJ, BasalMedia) and 10% FBS. All cells were incubated at 37 °C in a humidified environment with a CO_2_ concentration of 5%.

### Animals

The Ai6 (RCL-ZsGreen) mice were obtained from The Jackson Laboratory. 8-week-old male BALB/c and C57BL/6 mice were also utilized. All animal experiments were strictly conducted under pathogen-free conditions at the animal facility of the Shanghai Jiao Tong University School of Medicine. The animal protocol was reviewed and approved by the Institutional Animal Care and Use Committees of the School of Medicine, Shanghai Jiao Tong University.

### *In vitro* transcribed sPD-L1/Luc/Cre mRNA

To synthesize sPD-L1/Luc/Cre mRNA *in vitro*, a one-step method was employed. During PCR amplification with plasmid templates, poly(A) tails were added through primer extension. Following purification, the dsDNA products served as mRNA template fragments. The IVT mixture contained a DNA template, EZ-cap (B8177), ATP (K1043), CTP (K1045), or a chemically modified version, 5-methyl-CTP (B7967), GTP (K1044), UTP (K1048), or a chemically modified version, pseudo-UTP (B7972)/5-methoxy-UTP (B8061)/N_1_-methylpseudo-UTP (B8049), and T7 RNA Enzyme Mix (K1083). All these ingredients are purchased from APE×BIO. After incubating the mixture at 37 °C for 3 hours, the template was digested using DNase I (EN0521, ThermoFisher). Subsequently, purification was performed using the RNA Clean and Concentrator Kit (R1017, ZymoResearch). Then, cellulose purification was conducted following a previously established protocol to eliminate dsRNA 33.

### Preparation of mRNA-LNPs

The LNP formulations were prepared according to previously described methods 29. In brief, for mRNA-MC3-LNPs, lipids were dissolved in ethanol at molar ratios of 50:10:38.5:1.5 for DLin-MC3-DMA, DSPC, cholesterol, and PEG2000-DMG, respectively. For mRNA-DOTAP-LNPs, the molar ratios for DOTAP (890890, Avanti), DLin-MC3-DMA (A8791, APE×BIO), DSPC (850365, Avanti), cholesterol (B1702, APE×BIO), and PEG2000-DMG (880151, Avanti) were 50:25:5:19.2:0.8. The lipid mixture was combined with an equal volume of mRNA solution in 50 mM citrate buffer (pH 3.0) using a T-mixer. The formulations were immediately diluted 2-fold with 50 mM citrate buffer (pH 3.0), dialyzed in PBS (pH 7.4) using slide-a-lyzer dialysis cassettes (66380, ThermoFisher) for at least 15 h, and then concentrated using Amicon Ultra Centrifugal Filters (UFC8030, Millipore). The formulations were passed through a 0.22-μm filter (SLGP033RB, Millipore). RNA encapsulation was determined for all formulations using the Quant-iT RiboGreen RNA Assay (R11490, ThermoFisher), while particle sizes were analyzed using a nanoparticle tracking analysis instrument (Zetasizer Nano ZS, Malvern).

### Cryogenic transmission electron microscopy (Cryo-TEM)

The Cryo-TEM sample preparation and imaging procedures were conducted in the following manner: Firstly, a volume of 5 μL of the sample was meticulously applied onto a glow-discharged grid (R1.2/1.3 Au, 300 mesh, GiG). Subsequently, the grids were subjected to blotting for a duration of 4 seconds under 100% humidity at a temperature of 4 °C. Without delay, the grids were rapidly submerged into liquid ethane by employing a Mark IV vitrobot (ThermoFisher). The imaging process was performed utilizing a Talos Glacios transmission electron microscope (ThermoFisher). The magnification level was set to 92,000×, with a pixel size of 1.57 Å.

### Transfection of mRNA or mRNA-LNPs

To assess the expression efficiency of mRNA, cells were seeded in a 6-well plate until reaching 50%-70% confluency. Subsequently, transfection was performed using 2 μg of sPD-L1 mRNA in 4 μL of Lipofectamine 2000 (11668019, ThermoFisher), or 2 μg of sPD-L1 mRNA-LNPs.

### Western blot

Cells were lysed in mammalian cell lysis buffer (MCLB) containing 50 mM Tris (pH 7.5), 150 mM NaCl, 0.5% NP-40, and supplemented with EDTA-free protease inhibitor cocktail (04693159001, Roche) and 1 mM phenylmethyl sulfonyl fluoride (PMSF, 0754, Amresco). Protein concentrations were measured using the Bradford assay (5000201, Bio-Rad). Then, the protein was boiled in 5×sodium dodecyl sulfate sample buffer. The protein from each sample was separated by sodium dodecyl sulfate polyacrylamide gel electrophoresis and then transferred to a nitrocellulose membrane (Millipore). The membranes were blocked and incubated with primary antibodies (diluted 1:1000) in primary antibody dilution buffer (Beyotime Biotechnology). The primary antibodies used were anti-HA-Tag rabbit mAb (C29F4, CST), anti-GAPDH mAb (60004-1-Ig, Proteintech), and anti-PD-L1 mAb (ab205921, Abcam). The membranes were then incubated with goat anti-rabbit IgG (H+L) secondary antibody (31460, ThermoFisher) or goat anti-mouse IgG (H+L) secondary antibody (31430, ThermoFisher). Images were acquired using the ChampChemi imaging system (Sage Creation Science).

### Immunoprecipitation of sPD-L1 from supernatant

The cells were cultured until they reached 50%-70% confluence and then transfected with sPD-L1-HA mRNA. After 24 hours of transfection, the supernatant from the transfected cells was collected into a 15 mL centrifuge tube after passing through a 0.45-μm filter. To ensure sample integrity, an EDTA-free protease inhibitor cocktail (4693132001, Roche) was added. Subsequently, HA magnetic beads (88836, ThermoFisher) were added to the supernatant and incubated overnight at 4 °C with gentle rotation. On the following day, the immunocomplexes that precipitated onto the magnetic SampleRack were washed four times with ice cold MCLB. Finally, the samples were subjected to western blot for further analysis.

### Co-localization of mRNA-DOTAP-LNPs with lysosomes

During the *in vitro* transcription of sPD-L1 mRNA, 25% Cy3-UTP (B8330, APE×BIO) was incorporated. Subsequently, the fluorescent mRNA was encapsulated within DOTAP-LNPs. After 18 hours of LNPs transfection in cells, LysoTracker Green DND-26 (40738ES50, Yeasen) was added and incubated for 30 minutes. Live cell imaging was then performed using a Leica DMi8 S Live cell microscope.

### *In vivo* delivery of mRNA-LNPs or protein

mRNA-LNPs (0.2 mg/kg) or recombinant mouse PD-L1-Fc chimera protein (0.8 mg/kg) (758208, Biolegend) was administered via the tail vein. Blood samples were collected from the orbit at different time points using White's buffer (1:9) as an anticoagulant, and immediately centrifuged at 4,000 rpm for 10 minutes. The serum samples were then frozen at -80 °C. For detecting the abundance of PD-L1 protein in lung tissue, mouse lung tissue was collected at different time points after injection, placed in radioimmunoprecipitation assay lysis buffer (WB3100, NCM Biotech), frozen, ground, and then centrifuged to obtain protein. The protein abundance was analyzed using western blot.

### *In vivo* imaging of mRNA delivery

To investigate the biodistribution of LNPs in mice, we administered mRNA-MC3-LNPs or mRNA-DOTAP-LNPs expressing firefly luciferase (Luc) through the tail vein of BALB/c mice. At 6, 24, and 48 hours after LNPs injection, the mice were intraperitoneally injected with 150 mg/kg D-luciferin (122799, PerkinElmer). Ten minutes after the luciferin injection, IVIS Spectrum CT system (128201, PerkinElmer) were used to image the mice. We quantified luminescence values using Living Image software (PerkinElmer).

### Nebulization delivery of mRNA-LNPs

The nebulization delivery of mRNA-LNPs was carried out using the MicroSprayer Aerosolizer Model YAN 30012 (Yuyanbio) for targeted lung delivery. Each device was equipped with mounting quantitative columns, enabling precise measurement of 50 µL of the aerosolized solution. The aerosol droplets had a particle size of 30-50 μm. During the experimental procedure, mice were anesthetized with 4% isoflurane to ensure immobilization. The tongue was gently depressed using a tongue depressor to visualize the pharynx and facilitate the identification of the tracheal entrance. Subsequently, the nebulizer catheter was carefully inserted, and 50 µL of the aerosolized solution was rapidly delivered into the airway. To evaluate the nebulization efficiency, we conducted pre- and post-nebulization analysis of the mRNA encapsulated in LNPs using the Quant-iT RiboGreen RNA Assay (R11490, ThermoFisher). By comparing the measurements before and after nebulization, we accurately calculated the nebulization loss.

### Gene editing (Cre mRNA-DOTAP-LNPs) conducted in the Ai6 mice model

The Cre mRNA-DOTAP-LNPs were prepared as described above and administered via intravenous (i.v.) injections at a dosage of 0.8 mg/kg. After 2 days, the mice were euthanized, and their lung, liver, and spleen tissues were collected for flow cytometry analysis. Additionally, the left lung tissue was collected specifically for DAPI staining of freezing sections.

### Cell isolation and staining for flow cytometry

After euthanizing the mice, the lung tissue was immersed in 1640 medium, while the spleen and liver tissues were placed in high-glucose DMEM medium. For lung cell extraction, the lung tissue was minced and subjected to a 30-minute digestion at 37 °C, using type I collagenase (40507ES76, Yeasen) and DNase I (18047019, ThermoFisher). The digested tissue was then grinded and filtered through a 70-mesh nylon mesh to obtain a single-cell suspension. For spleen cell extraction, the minced spleen tissue was directly grinded and filtered through a 70-mesh nylon mesh. For liver cell extraction, the liver tissue was transferred into the type II collagenase buffer (type II collagenase: LS004176, Worthington). It was then digested at 37 °C for 10 minutes and filtered through a 70 μm mesh filter (CSS025070, BIOFIL) into a new tube.

The collected tissue single-cell suspensions were treated with red blood cell lysis buffer after centrifuging (C3702, Beyotime). Following PBS resuspension, the cells were incubated with FVS780 viability dye (565388, BD Horizon) for 15 minutes. After one wash with Fluorescence-Activated Cell Sorting buffer (FACS, PBS with 1% FBS), CD16/32 Fc block (553142, BD Pharmingen) was added and incubated for 20 minutes. Subsequently, flow cytometry antibodies were added and incubated at 4 °C for 30 minutes. After another wash with FACS buffer, the samples were analyzed using flow cytometry (Beckman Coulter CytoFLEX LX). The lung tissue was stained with PerCP/Cy5.5-CD45 antibody (564106, BD Pharmingen), BV421-F4/80 antibody (565411, BD Horizon), PE-CD31 antibody (553373, BD Horizon), and APC-CD326 antibody (17-5791-82, ThermoFisher). The gating strategy and data analysis were performed using CytExpert software, as illustrated in Figure S3.

### Freezing section DAPI staining and confocal microscopy imaging

The lung tissue was fixed by freezing it in optimal cutting temperature (OCT) compound (G6059, Servicebio), and thin sections were sliced and mounted onto glass slides. The frozen slices were then allowed to warm up to room temperature, and the drawing circles were organized. The slides were washed three times by shaking them in PBS. Subsequently, DAPI dye (G1012, Servicebio) was added and incubated at room temperature for 10 minutes, protected from light. The slides were then washed again. Finally, anti-fluorescence quenching sealing tablets (G1401, Servicebio) were applied to the slides to complete the staining process. The sections were imaged using the confocal microscopy (Leica STELLARIS 8 FALCON FLIM Microscope). Due to the pronounced green autofluorescence in lung tissue, we employed the fluorescence lifetime imaging module of the microscope to record the green fluorescence signal with a lifetime of 2.6 ns, aiming to eliminate the interference caused by autofluorescence.

### *In vivo* evaluation of sPD-L1 mRNA expression

Serum samples were diluted 1/300 with reagent diluent and tested using the Mouse PD-L1 DuoSet ELISA kit (DY1019-05, R&D Systems) following the manufacturer's protocol. OD450 and OD570 were measured using a microplate reader (Synergy H1, BioTek). The standard sample data were recorded in Prism and fitted with a 4PL curve (x represents the concentration) to calculate the sample concentration based on the curve.

### Preparation of the ARDS model

Monoclonal *Pseudomonas aeruginosa* (PAO) was cultured in Luria-Bertani medium supplemented with spectinomycin (1:1000) and shaken for 12 hours at 37 °C and 220 rpm. The bacterial solution was then resuspended in PBS and adjusted to contain 2×10^6^ bacterial colony-forming units (CFU) after centrifugation at 3000 rpm for 20 minutes. An intraperitoneal injection of 4% chloral hydrate (0.1 mg/10g) was used to anesthetize the mice and fix them on the surgical plate. Endotracheal intubation was performed using a guide wire, and 40 μL of the PAO bacterial solution was instilled into the lungs.

### Pathology assessment of ARDS mouse

To euthanize the mice, the cervical spine was severed, and the chest was opened to expose the lungs. The right lung was ligated, and the left lung was flushed with PBS to collect the bronchoalveolar lavage fluid (BALF). The BALF was centrifuged at 500×g for 5 minutes, and the supernatant was separated from the cell pellet. The cell pellet was then resuspended in red blood cell lysis buffer and subsequently resuspended in 0.2 mL of PBS for the BALF white blood cell count using a cell counter (JIMBIO). The protein concentration of the BALF supernatant was measured using the Bradford method, and the absorbance was read using a microplate reader (Synergy H1, BioTek). The lower lobe of the right lung was minced to obtain a single-cell suspension for flow cytometry, while the upper lobe was used for pathological sections or wet-to-dry weight ratio assay. For pathological sections, the lung tissue was fixed with 4% paraformaldehyde, embedded in paraffin, and sectioned and stained with hematoxylin and eosin (H&E). The freshly obtained right upper lobe of the mouse lung was weighed immediately and recorded as the wet weight. The sample was then placed in a 50 °C oven and dried for 48 hours before being weighed again and recorded as the dry weight. The wet-to-dry weight ratio was used to measure the degree of lung tissue edema.

### Flow cytometry test of lung immune cells

The cell suspension of was collected as mentioned above. The cell pellet was dyed with FVS570 (564995, BD Pharmingen) and resuspended in FACS, added with CD16/32 (553142, BD Pharmingen), and incubated at 4 °C for 15 minutes. Then we performed flow cytometry staining using BV421-CD279 antibody (135217, Biolegend) and APC-CD45 antibody (559864, BD Pharmingen). We analyzed the cells using the Beckman CytoFlex S machine.

### Lung injury scoring

To ensure a comprehensive evaluation of the severity of histological lung injuries, we adopted a systematic approach by randomly selecting two specific fields of view from each hematoxylin and eosin (HE)-stained histological section. Subsequently, these selected fields underwent a previously reported semi-quantitative assessment (Table 1) 34.

### Measurement of survival rate

Seventy-five C57BL/6 mice were divided into five groups, and four groups underwent ARDS modeling through intratracheal instillation of PAO. Four hours later, the groups were separately intravenously injected with the following agents in equal volumes: PBS, 0.8 mg/kg PD-L1-Fc, 0.2 mg/kg of sPD-L1 mRNA-MC3-LNPs, and 0.2 mg/kg of sPD-L1 mRNA-DOTAP-LNPs. The survival rates of the mice were monitored daily for a 7-day period after treatment with the respective agents.

### Comparison of bacterial clearance *in vivo*

After establishing the PAO-induced ARDS mouse model, mice were administered tail vein injections of either PBS or sPD-L1 mRNA-DOTAP-LNPs (0.2 mg/kg) at 4 hours post-PAO. After a 12-hour interval, BALF was collected from the mice and diluted before being plated on agar plates. Subsequently, the agar plates were incubated at 37 °C for one day to facilitate the assessment of bacterial colony counts.

### Evaluation of immune cell populations in mouse spleen and thymus

Twelve mice were divided into four groups to receive different treatments via tail vein injection as follows: PBS, PD-L1-Fc (0.8 mg/kg), sPD-L1 mRNA-MC3-LNPs (0.2 mg/kg), and sPD-L1 mRNA-DOTAP-LNPs (0.2 mg/kg). After a 3-day period, the mice were euthanized, and their spleen tissues were immersed in 1640 culture medium, while the thymus tissues were immersed in high-glucose DMEM. The spleen tissues were processed to obtain cell suspensions, following the previously described method. Thymus cell extraction involved mincing the thymus tissues and treating them with type IV collagenase (40510ES60, Yeasen) and DNase I (18047019, ThermoFisher), followed by digestion at 37 °C for 20 minutes. Subsequently, single-cell suspensions were obtained by passing the thymus cells through a 70-mesh nylon mesh.

The obtained cell suspensions were then treated with red blood cell lysis buffer and dyed with FVS510 viability dye (423102, Biolegend) for 15 minutes. To prevent non-specific binding, CD16/32 Fc block (553142, BD Pharmingen) was used for blocking. Following that, the cell suspensions were incubated at 4 °C for 30 minutes with the following flow cytometry antibodies: APC-CD86 antibody (558703, BD Pharmingen), PerCP/Cy5.5-MHC II antibody (107626, Biolegend), APC/Cy7-CD11c antibody (117324, Biolegend), FITC-CD4 (553046, BD Pharmingen), PE/Cy7-CD8a antibody (100721, Biolegend), and PE-CD25 antibody (553075, BD Pharmingen). After another wash with FACS buffer, the samples were analyzed using flow cytometry (Beckman Coulter CytoFLEX LX). The gating strategy and data analysis were performed using CytExpert software, as illustrated in Figure S6.

### Statistical analysis

All statistical analyses and graphs were performed using GraphPad Prism 8 software, and two-sided t-tests were used. The error bars represent the standard error of the mean (SEM), unless otherwise specified as the standard deviation (SD). Statistical significance was set at p < 0.05, and the level of significance in figures is represented as follows: *p < 0.05, **p < 0.01, ***p < 0.001, and ****p < 0.0001.

## Results

### Expression of sPD-L1 mRNA-LNPs *in vitro*

**Figure 1:**
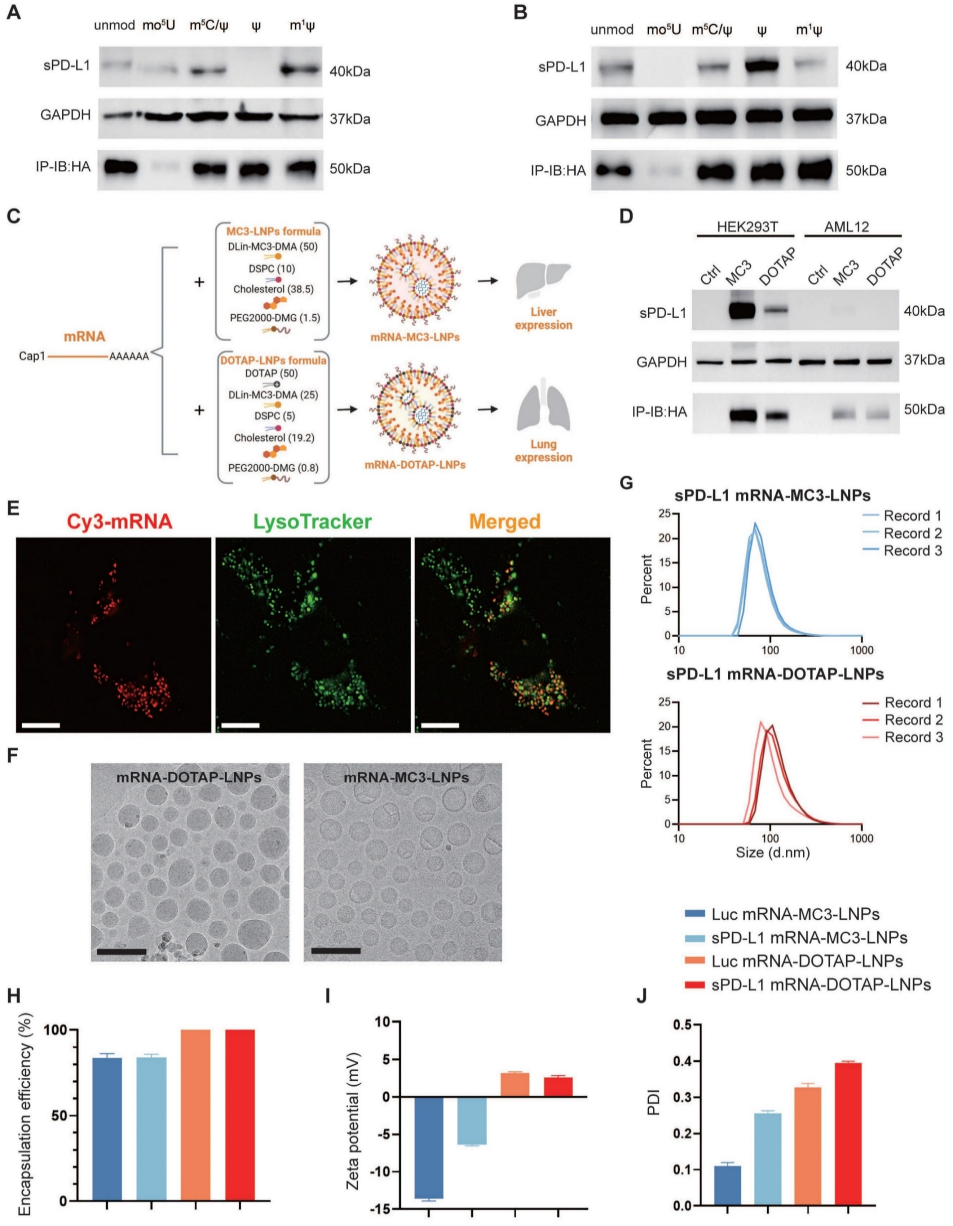
** sPD-L1 mRNA expression *in vitro* and physiochemical characterization of mRNA-MC3-LNPs and mRNA-DOTAP-LNPs.** (A-B) Expression of HA-tagged sPD-L1 mRNA with different nucleotide modifications in HEK293T (A), AML12 (B). Unmod, nucleotides without any modification; mo^5^U, 5-methoxyuridine; m^5^C/ψ, 5-methylcytosine and pseudouridine; ψ, pseudouridine; m^1^ψ, N_1_-methylpseudouridine. IP-IB: HA, using anti-HA magnetic beads to immunoprecipitate the sPD-L1-HA protein in cell culture supernatant and immunoblotting was detected with the anti-HA antibody. (C) Formulation of mRNA-MC3-LNPs and mRNA-DOTAP-LNPs. Percentages in parentheses show the molar ratios of the ingredients. DSPC, 1,2-distearoyl-sn-glycero-3-phosphocholine; PEG, polyethylene glycol; DOTAP, 1,2-dioleoyl-3-trimethylammonium-propane. (D) Expression of HA-tagged sPD-L1 mRNA-LNPs with m^1^ψ modification in HEK293T and AML12. (E) Representative image depicting the co-localization of sPD-L1 mRNA-DOTAP-LNPs (25% Cy3-UTP modified) with lysosomes (FITC-lysotracker) in A549 cells 18 hours post-transfection. Scale bar, 10 μm. (F) Cryo-TEM images of sPD-L1 mRNA-DOTAP-LNPs and mRNA-MC3-LNPs. Scale bar, 200 nm. (G) Particle size distribution of sPD-L1 mRNA-MC3-LNPs and mRNA-DOTAP-LNPs. Each data was recorded 3 times. d.nm, diameter (nm). (H) Encapsulation efficiency of Luc/sPD-L1 mRNA-MC3-LNPs and mRNA-DOTAP-LNPs (n = 3). (I) Zeta potential of Luc/sPD-L1 mRNA-MC3-LNPs and mRNA-DOTAP-LNPs. Results represent mean ± SEM (n = 3). (J) Polydispersity index (PDI) of Luc/sPD-L1 mRNA-MC3-LNPs and mRNA-DOTAP-LNPs. Results represent mean ± SEM (n = 3).

As PD-L1 is an immune inhibitory molecule, our objective is to utilize mRNA-LNPs technology for the safe and reliable expression of a soluble form of PD-L1 (sPD-L1). To determine the most efficient chemical modification of *in vitro* transcribed (IVT) mRNA, we conducted western blot assay to assess the expression efficiency of HA tagged sPD-L1 mRNA using five different chemical nucleotide modifications in HEK293T cells and a mouse liver cell line AML12. The five modifications tested were as follows: unmodified (unmod), 5-methoxyuridine (mo^5^U), 5-methylcytosine and pseudouridine (m^5^C/ψ), pseudouridine (ψ), and N_1_-methylpseudouridine (m^1^ψ). Our analysis revealed the presence of a 40 kDa precursor protein in the whole cell lysate, and a 50 kDa protein in the supernatant (Figure 1A-B). Notably, we observed that the sPD-L1 protein expressed by mRNA modified with N_1_-methylpseudo-UTP (m^1^ψ) exhibited the highest abundance in the cell culture medium (Figure 1A-B), which aligns with our objective of maximizing sPD-L1 release into the extracellular space. Additionally, the m^1^ψ modification has the potential to decrease immunogenicity and reduce dsRNA production 33, 35. Therefore, we selected m^1^ψ-modified sPD-L1 mRNA for encapsulation in LNPs for future studies.

Considering the inherent instability and susceptibility to degradation of mRNA, we opted to employ LNPs as carriers to deliver sPD-L1 mRNA into the body. To achieve lung tissue-specific immunosuppression, we incorporated selective organ targeting (SORT) technology, which facilitates precise engineering of nanoparticles for targeted mRNA delivery 29. mRNA-MC3-LNPs as traditional LNPs primarily accumulate in the liver, leading to the release of the expressed soluble protein into the circulatory system. In contrast, mRNA-DOTAP-LNPs (50% DOTAP) are specifically designed to target the lungs when administered via intravascular injection. To compare *in situ* expression with systemic expression of sPD-L1, we encapsulated sPD-L1 mRNA in both types of LNPs (Figure 1C). To confirm successful loading of sPD-L1 mRNA into LNPs, we tested and assessed its expression in cultured cells. Our findings indicated that the delivery of mRNA-DOTAP-LNPs resulted in an overall lower expression level compared to mRNA-MC3-LNPs delivery (Figure 1D). LNP uptake has been reported to depend on receptor-mediated endocytosis, followed by endosomal and lysosomal fusion 36, 37. To investigate the uptake mechanism of DOTAP-LNPs, we synthesized Cy3-fluorescent-labeled mRNA *in vitro* and encapsulated it within DOTAP-LNPs. Transfection was conducted on the A549 lung cancer cell line. After 18 hours, we observed co-localization of mRNA with lysosomes, providing evidence that DOTAP-LNPs also enter cells through endocytosis (Figure 1E).

### Physiochemical characterization of mRNA-MC3-LNPs and mRNA-DOTAP-LNPs

To assess the physicochemical properties of the LNPs, we conducted measurements of the encapsulation efficiency, size, and zeta potential of both types of mRNA-LNPs. The LNPs were subjected to morphological analysis using transmission electron microscopy (TEM), which provided insight into their distinct shapes and sizes (Figure 1F). Determination of protein size utilizing Malvern Zetasizer Nano ZS confirmed that both mRNA-MC3-LNPs and mRNA-DOTAP-LNPs exhibited similar size distribution, with diameters around 100 nm (Figure 1G, Figure S1). Furthermore, mRNA-DOTAP-LNPs demonstrated a high encapsulation efficiency, reaching up to 100% (Figure 1H), and exhibited a positive charge with a zeta potential ranging from 2-4 mV (Figure 1I). On the other hand, mRNA-MC3-LNPs displayed an encapsulation efficiency of approximately 85% and carried a negative charge within the range of -15 to -5 mV (Figure 1H-I). mRNA-MC3-LNPs displayed a polydispersity index (PDI) of 0.1-0.3, whereas mRNA-DOTAP-LNPs exhibited a slightly higher PDI ranging from 0.3 to 0.4 (Figure 1J).

### Expression and biodistribution of mRNA-DOTAP-LNPs

**Figure 2:**
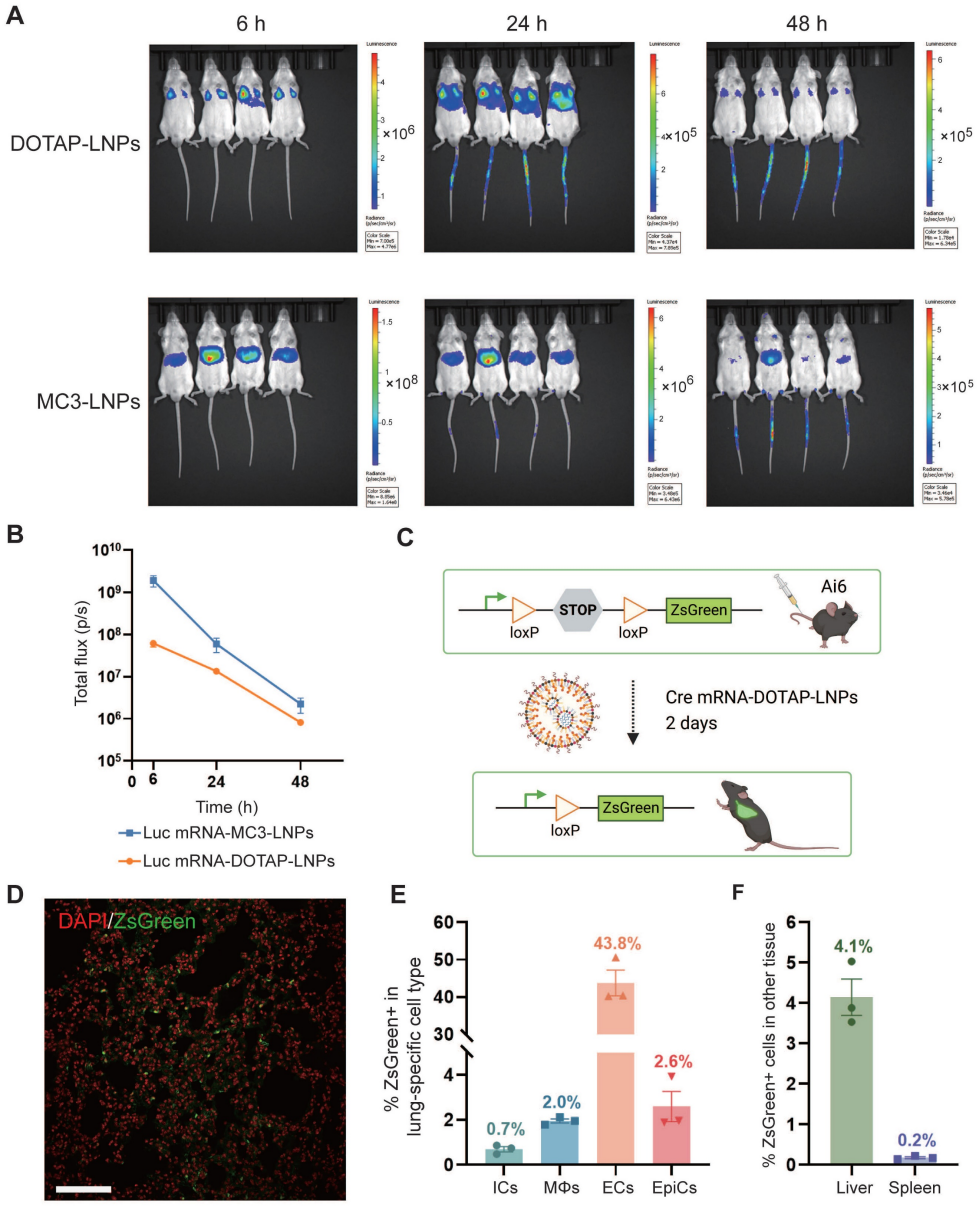
** Pulmonary cells were targeted by mRNA-DOTAP-LNPs.** (A) Representative whole-body bioluminescence images of mice at different time points (6 h, 24 h, 48 h) after intravenous administration of 0.4 mg/kg Luc mRNA-MC3-LNPs or mRNA-DOTAP-LNPs. The scale of luminescence is different for the different time points and groups. (B) Quantification of bioluminescence intensity of (A). Results represent mean ± SEM (n = 4). (C) Diagram illustrating the delivery of Cre mRNA using DOTAP-LNPs to activate ZsGreen expression in Ai6 transgenic mice. (D) Representative confocal image of the DAPI-stained ZsGreen+ lung section. Scale bar, 50 μm. (E) Percentage of ZsGreen+ cells within defined cell type populations in the lung measured by flow cytometry. Immune cells (ICs) were stained with PerCP/Cy5.5-CD45 antibody. Macrophages (MΦs) were stained with BV421-F4/80 antibody. Endothelial cells (ECs) were stained with PE-CD31 antibody and epithelial cells (EpiCs) were stained with APC-CD326 antibody. Results are represented as mean ± SEM (n = 3). (F) Percentage of ZsGreen+ cells in the liver and spleen. Results represent mean ± SEM (n = 3).

To investigate the tissue-specific delivery of these LNPs *in vivo*, Balb/c mice were administered luciferase mRNA, encapsulated within both types of LNPs, via intravenous (i.v.) injection at a dosage of 0.4 mg/kg. The expression of luciferase protein was assessed at various time points (6 h, 12 h, 24 h, 48 h) after intraperitoneal administration of the substrate Luciferin, which generated a robust bioluminescent signal corresponding to the expression level. As anticipated, delivery of luciferase mRNA via mRNA-DOTAP-LNPs predominantly resulted in expression in the lungs, while mRNA-MC3-LNPs facilitated significant expression in the liver (Figure 2A). The bioluminescent signal in the targeted organs peaked at 6 hours after LNPs injection and remained detectable for over 48 hours (Figure 2A). Quantitative analysis of luminescence values revealed that the expression level of luciferase mRNA delivered by mRNA-MC3-LNPs in the liver was more than 10-fold higher than that of mRNA-DOTAP-LNPs in the lungs at the 6-hour time point. As time progressed, the signal intensity decayed by over 100-fold within 48 hours (Figure 2B). Furthermore, we conducted experiments to explore the possibility of utilizing nebulization for LNP delivery. However, in assays to detect bioluminescent signals following luciferase mRNA delivery, we observed that the lung tissue expression level after 6 hours was significantly lower, approximately 100-fold, compared to the intravenous administration of DOTAP-LNPs (Figure S2A-B). Moreover, the aerosol delivery method led to a substantial loss of mRNA-LNPs, with a loss of over 30% due to the aerosolizer (Figure S2C). Due to these limitations, we decided not to proceed with nebulization as a viable method for lung-targeted delivery.

To identify specific lung cell subpopulations transfected by DOTAP-LNPs, we utilized a genetically engineered Cre/LoxP Ai6 reporter mouse line, allowing for Cre drove specific expression of the ZsGreen protein 38. We administered 0.8 mg/kg Cre mRNA-DOTAP-LNPs to Ai6 transgenic mice (Figure 2C). After 2 days, ZsGreen fluorescence signals were observed through confocal microscopy (Figure 2D). Subsequently, we performed flow cytometry analysis on lung cell suspensions, utilizing specific cell type markers. The results revealed that 0.7% of immune cells (ICs) and 2% of macrophages (MΦs) were transfected, while 43.8% of endothelial cells (ECs) exhibited ZsGreen fluorescence, and the percentage for epithelial cells (EpiCs) was 2.6% (Figure 2E, Figure S3). Additionally, we examined the expression of ZsGreen fluorescent protein in the liver and spleen. Our findings demonstrated that 4.1% of liver cells and 0.2% of spleen cells were transfected (Figure 2F).

### Expression of sPD-L1 mRNA-MC3-LNPs and sPD-L1 mRNA-DOTAP-LNPs *in vivo*

**Figure 3:**
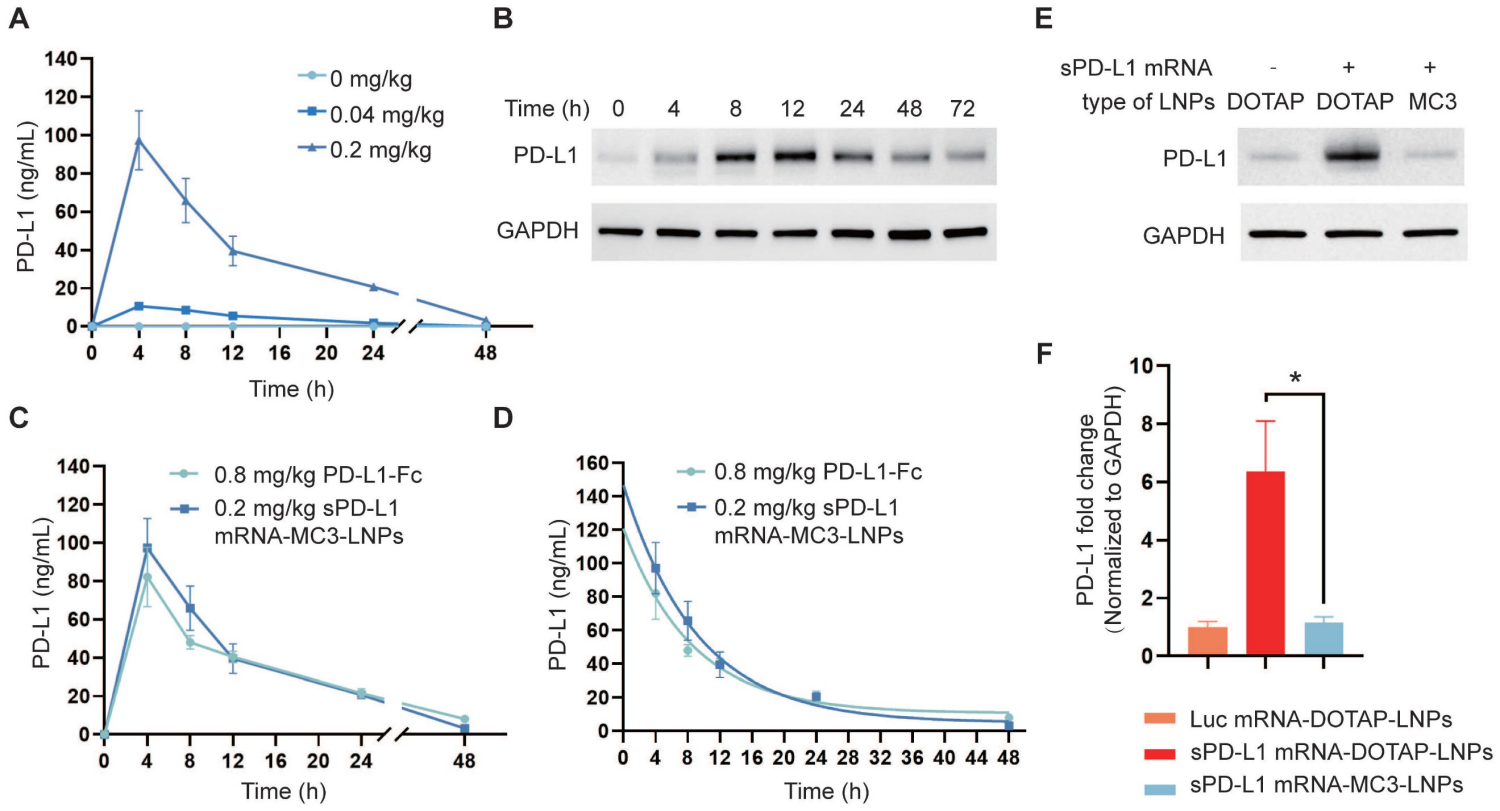
** Expression of sPD-L1 mRNA-MC3-LNPs and mRNA-DOTAP-LNPs *in vivo*.** (A) PD-L1 levels in circulatory system over time (0 h, 4 h, 8 h, 12 h, 24 h, 48 h) after intravenous injection of sPD-L1 mRNA-MC3-LNPs (0, 0.04 or 0.2 mg/kg). (B) PD-L1 levels in lung tissue over time (0 h, 4 h, 8 h, 12 h, 24 h, 48 h) after intravenous injection of sPD-L1 mRNA-DOTAP-LNPs (0.2 mg/kg). The expression level of PD-L1 in the lysate of lung tissue were valued by western blot. (C) Blood concentration of sPD-L1 after intravenously injection of sPD-L1 mRNA-MC3-LNPs (0.2 mg/kg) or PD-L1-Fc recombinant protein (0.8 mg/kg) into mice (n = 4), and blood samples were collected at different time points (0 h, 4 h, 8 h, 12 h, 24 h, 48 h) to determine protein expression by ELISA kit. (D) The half-life of sPD-L1 mRNA-MC3-LNPs (0.2 mg/kg) and PD-L1-Fc chimera (0.8 mg/kg). (E) Expression level of PD-L1 in lung tissue detected by western blot assay. The tissues were collected at 4 hours after intravenous injection of Luc mRNA-DOTAP-LNPs (0.2 mg/kg), sPD-L1 mRNA-DOTAP-LNPs (0.2 mg/kg) or sPD-L1 mRNA-MC3-LNPs (0.2 mg/kg). (F) Comparison of the PD-L1 abundance in lung tissue between groups. Results represent mean ± SEM (n = 3). *p < 0.05. t-test analysis.

To investigate the translation of sPD-L1 LNPs into sPD-L1 protein following intravenous administration, we administered both LNPs to C57BL/6 mice via i.v. injection at a dosage of 0.2 mg/kg. It is known that soluble proteins expressed in the liver are released into the bloodstream and transported to sites of inflammation in lung tissue through circulation 39. Therefore, we monitored the levels of sPD-L1 in the plasma over time (0 h, 4 h, 8 h, 12 h, 24 h, 48 h) using an ELISA assay following sPD-L1 mRNA-MC3-LNPs injection. The results demonstrated a dose-dependent effect of sPD-L1 mRNA-MC3-LNPs, with a dose of 0.2 mg/kg resulting in a plasma protein level of 100 ng/mL. The expression level reached its peak at 4 hours post-injection and gradually declined within 48 hours (Figure 3A). In contrast, we did not detect sPD-L1 protein in the plasma following sPD-L1 mRNA-DOTAP-LNPs administration (results not shown). Subsequently, we extracted lung tissue protein at different time points (0 h, 4 h, 8 h, 12 h, 24 h, 48 h, 72 h) after injection and assessed the abundance of PD-L1 using western blot analysis. We observed that the highest abundance was observed at 8-12 hours, and the expression of sPD-L1 was sustained for over 72 hours (Figure 3B). These results indicated that the protein expression from mRNA delivered by DOTAP-LNPs is localized rather than systemic.

PD-L1-Fc recombinant protein is a glycosylated protein consisting of the extracellular domain of PD-L1 linked to the fragment crystallizable region (Fc) of mouse immunoglobulin G1 (IgG1). The fusion of Fc segment can reduce complement activation effects and increase the protein's half-life to prolong circulation time 40. This PD-L1-Fc recombinant protein has been demonstrated to effectively alleviate acute immune reactions 19. To compare the therapeutic effects of protein and mRNA-LNPs, we also evaluated the half-life of PD-L1-Fc recombinant protein following i.v. injection into the body at a dosage of 0.8 mg/kg. We compared the plasma levels and half-life of sPD-L1 expressed by PD-L1-Fc and sPD-L1 mRNA-MC3-LNPs in the mouse serum after i.v. injection at various time points (0 h, 4 h, 8 h, 12 h, 24 h, 48 h). Our findings revealed that the half-life curve of sPD-L1 mRNA-MC3-LNPs at a dosage of 0.2 mg/kg was similar to that of PD-L1-Fc at a dosage of 0.8 mg/kg (Figure 3C-D).

In the context of alleviating ARDS, localized expression of sPD-L1 in the lungs is crucial. Therefore, we compared the differences in target protein expression in lung tissue between the two LNPs. After extracting protein from lung tissue and conducting western blot analysis, we compared the levels of sPD-L1 delivered to lung tissue by mRNA-DOTAP-LNPs and mRNA-MC3-LNPs, also used Luc mRNA-DOTAP-LNP as a control. We observed that the expression level of sPD-L1 delivered by mRNA-MC3-LNPs was relatively low in lung tissue. However, *in situ* expression of sPD-L1 facilitated by mRNA-DOTAP-LNPs resulted in a 6-fold higher expression compared to endogenous PD-L1 (Figure 3E-F). The Luc mRNA-DOTAP-LNPs demonstrated a low level of PD-L1, suggesting that the increase in PD-L1 expression was specifically caused by sPD-L1 mRNA rather than non-specific SORT mRNA-LNPs (Figure 3E-F). This outcome indicates that the use of sPD-L1 mRNA-DOTAP-LNPs can significantly increase the expression level of sPD-L1 in lung tissue.

### PAO-induced ARDS mouse models

**Figure 4:**
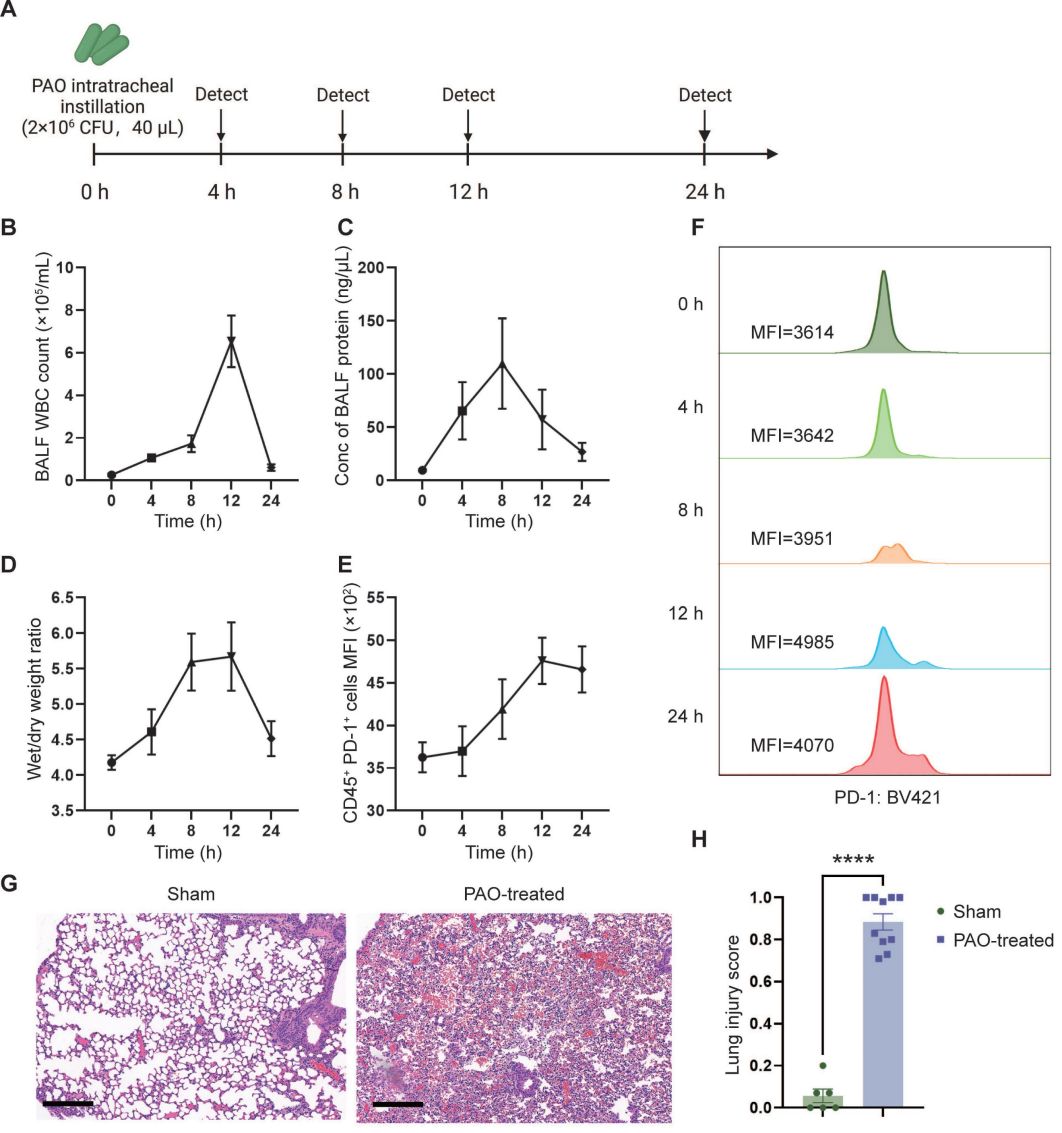
** PAO-induced ARDS mouse models.** ARDS mice were modeled by pouring PAO bacterial solution into the lungs by endotracheal intubation (2×10^6^ CFU/mL, 40 µL/mouse). ARDS mice were sacrificed at different time points (0 h, 4 h, 8 h, 12 h, 24 h) after modeling and bronchoalveolar lavage fluid (BALF) and partial lung tissue were collected for testing. (A) Schematic depicting the experiment workflow. (B) Time course of the white blood cell (WBC) count in the BALF. WBC were counted by Cell Count. Results represent mean ± SEM (n = 5). (C) Time course of the concentration of BALF protein. Concentration of protein was determined by Bradford method. Results represent mean ± SEM (n = 5). (D) Time course of wet/dry weight ratio of the right upper lobe of the lung. Wet weight of the tissues was weighed immediately and dry weight data were collected after 48 hours of drying. Results represent mean ± SEM (n = 3). (E) Time course of changes in PD-1 expression in lung immune cells (CD45^+^) over time. PD-1 was stained with BV421-CD279 antibody and CD45 was stained APC-CD45 antibody. Results represent mean ± SEM (n = 5). MFI, mean fluorescence intensity. (F) Histogram of PD-1 MFI at different time points. (G) Representative pathology of normal lung (left) and ARDS lung (right, at 12-hour time point). Scale bar, 200 μm. (H) Lung injury scoring comparison between groups with or without PAO treatment (n = 5). ****p < 0.0001.

To evaluate the therapeutic effect of sPD-L1 mRNA-LNPs, we initially established and assessed a mouse model of ARDS. To mimic human ARDS disease, we non-invasively administered *Pseudomonas aeruginosa* (PAO) to mice via intratracheal instillation (2×10^6^ CFU/mL, 40 µL/mouse) to induce symptoms similar to those observed in human bacterial infection-induced ARDS. To assess the severity and acute response of lung injury, we measured white blood cell (WBC) counts and protein concentration in the bronchoalveolar lavage fluid (BALF), as well as evaluated the wet/dry weight (indicative of edema) of the mouse lung at different time points (0 h, 4 h, 8 h, 12 h, 24 h) (Figure 4A). WBC count, protein concentration, and wet/dry weight showed a rapid increase following PAO administration, reaching their peaks at 8-12 hours (Figure 4B-D). Furthermore, the expression of PD-1, the receptor for PD-L1, began to rise at 4 hours and reached its peak at 12 hours post-PAO administration (Figure 4E-F). Histological analysis of lung inflammation at 12 hours after PAO administration confirmed the presence of increased protein and red blood cell accumulation, alveolar wall thickening, and immune cell recruitment (Figure 4G). To assess the severity of lung injury in mice after PAO modeling, we conducted a lung injury scoring based on the ARDS animal scale, using HE-stained lung tissue sections obtained before and after the modeling. The scoring unequivocally confirmed the presence of severe lung injury in mice following PAO modeling (Figure 4H, Figure S4).

### sPD-L1 mRNA-MC3-LNPs attenuated pulmonary inflammatory response in ARDS mice

**Figure 5:**
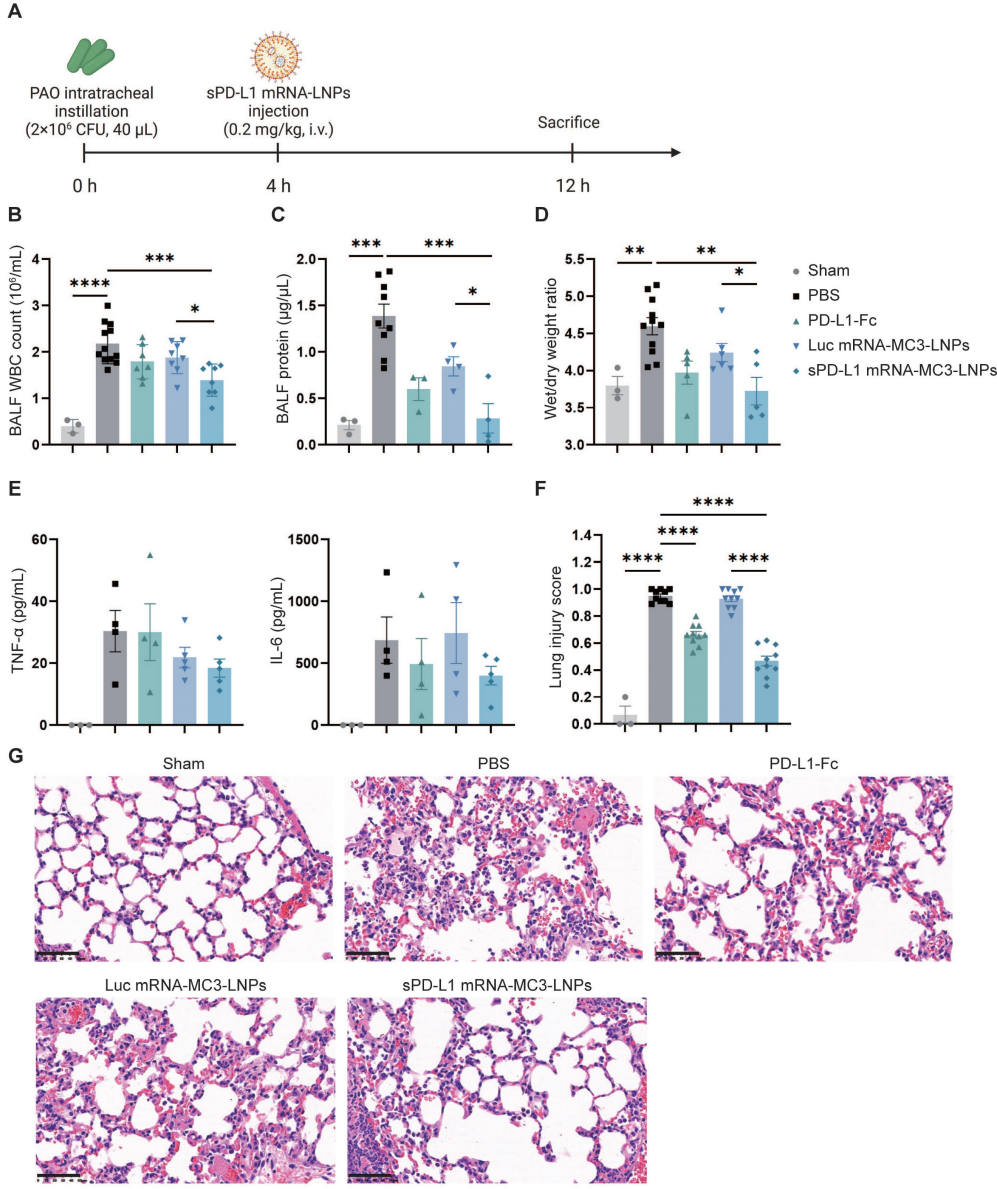
***In vivo* therapeutic effect of sPD-L1 mRNA-MC3-LNPs.** (A) Schematic depicting the experiment workflow. ARDS mice were modeled by pouring PAO bacterial solution into the lungs by endotracheal intubation (2×10^6^ CFU/mL, 40 µL/mouse). Four hours later, mice received a tail vein administration (PBS, 0.8 mg/kg PD-L1-Fc, 0.2 mg/kg Luc mRNA-MC3-LNPs, 0.2 mg/kg sPD-L1 mRNA-DOTAP-LNPs) (n = 3-12). After 12 hours, mice were sacrificed by cervical dislocation. Then BALF and partial lung tissue were collected for testing. Sham, use PBS solution to model. (A) Schematic depicting the experiment workflow. (B) WBC count in the BALF. WBC were counted by Cell Count. Results represent mean ± SEM (n = 3-12). (C) Protein concentration in the BALF. Concentration of protein was determined by Bradford methods. Results represent mean ± SEM (n = 3-9). (D) Wet/dry weight ratio of the right upper lobe. Wet weight of the tissue was weighed immediately and dry weight data were collected after 48 hours of drying. Results represent mean ± SEM (n = 3-11). (E) Concentration of inflammatory factor (TNF-α, IL-6) in the BALF. ELISA was used to determine the concentrations of the inflammatory cytokines. Results represent mean ± SEM (n = 3-5). (F) Lung injury scoring of HE stained sections (n = 3-5). *p < 0.05, **p < 0.01, ***p < 0.001, ****p < 0.0001. t-test analysis. (G) Representative pathology of ARDS lungs treated with different drugs (Sham, PBS, PD-L1-Fc, Luc mRNA-MC3-LNPs, sPD-L1 mRNA-MC3-LNPs, at 12-hour time point). Scale bar, 50 μm.

To assess the therapeutic effectiveness of sPD-L1 mRNA-MC3-LNPs, we administered i.v. injections of PBS, PD-L1-Fc protein (0.8 mg/kg), Luc mRNA-MC3-LNPs (0.2 mg/kg), and sPD-L1 mRNA-MC3-LNPs (0.2 mg/kg) four hours after intratracheally administering PAO to induce ARDS. At the 12-hour time point, we measured crucial inflammatory indicators, including WBC and protein concentration in the BALF. This evaluation enabled us to evaluate the efficacy of sPD-L1 mRNA-MC3-LNPs during the peak of the inflammation response observed in prior experiments (Figure 5A). Remarkably, compared to the PBS group or the Luc mRNA-MC3-LNPs control group, sPD-L1 mRNA-MC3-LNPs demonstrated significant effectiveness in reducing WBC count and protein concentration in BALF (Figure 5B-C), thereby alleviating pulmonary edema as reflected by the reduction of wet/dry weight ratio (Figure 5D). Additionally, we measured the levels of the inflammatory cytokines TNF-α and IL-6 in BALF and observed a slight downregulation following the administration of sPD-L1 mRNA-MC3-LNPs (Figure 5E). Furthermore, we conducted scoring of lung injury, and histological staining provided morphological evidence of the mitigated inflammatory pathology (Figure 5F). Following the induction of ARDS, the administration of sPD-L1 mRNA-MC3-LNPs demonstrated effective reduction in the presence of immune cells within the alveolar cavity and diminished blood leakage at the 12-hour time point (Figure 5G).

### Compared to sPD-L1 mRNA-MC3-LNPs, sPD-L1 mRNA-DOTAP-LNPs enhanced therapeutic efficacy and improved survival in ARDS

**Figure 6:**
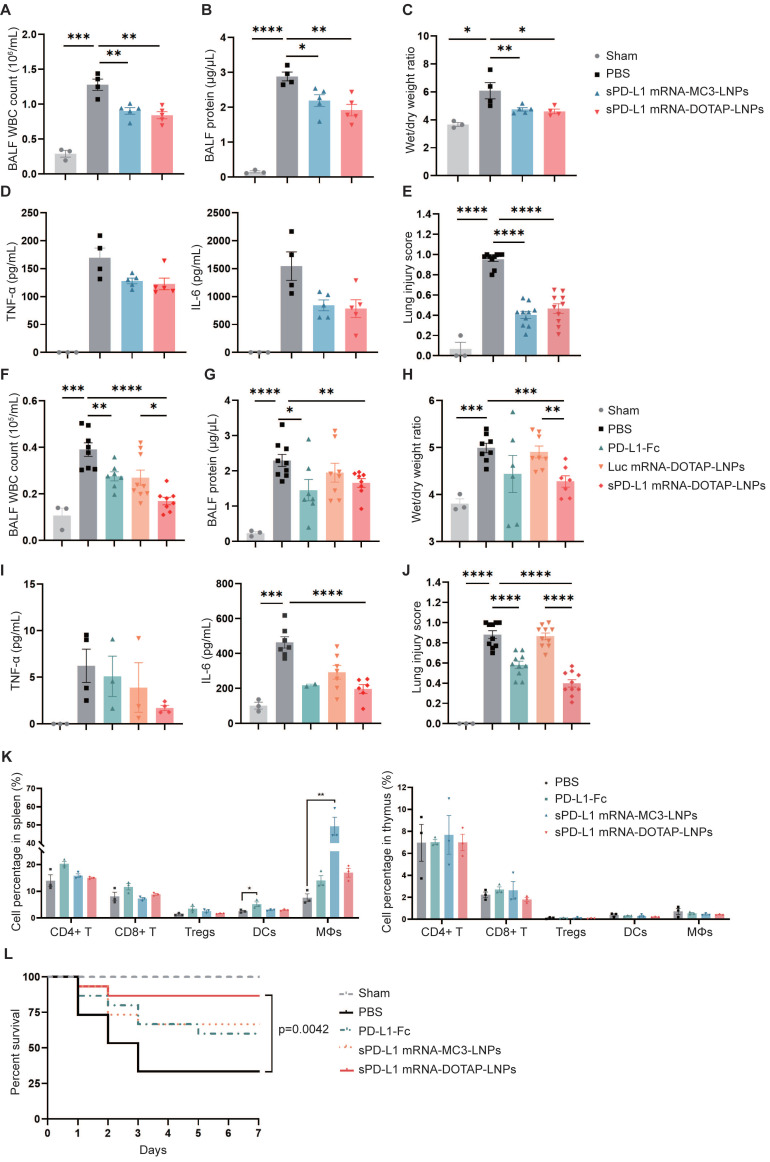
***In vivo* therapeutic effect of sPD-L1 mRNA-DOTAP-LNPs.** (A-E) Compare the therapeutic effect of PD-L1-Fc (0.8 mg/kg), sPD-L1 mRNA-MC3-LNPs and sPD-L1 mRNA-DOTAP-LNPs (0.2 mg/kg) by WBC count (A), protein concentration (B), wet/dry weight ratio of the right upper lobe (C), concentration of inflammatory factor TNF-α/IL-6 in the BALF (D), and lung injury scoring of HE stained sections (E). Results represent mean ± SEM (n = 3-5). *p < 0.05, **p < 0.01, ***p < 0.001, ****p < 0.0001. t-test analysis. (F-J) Test the therapeutic effect of sPD-L1 mRNA-DOTAP-LNPs (0.2 mg/kg) by WBC count (F), protein concentration (G), wet/dry weight ratio of the right upper lobe (H), concentration of inflammatory factor TNF-α/IL-6 in the BALF (I), and lung injury scoring of HE stained sections (J). Results represent mean ± SEM (n = 3-8). *p < 0.05, **p < 0.01, ***p < 0.001, ****p < 0.0001. t-test analysis. (K) Percentage of different immune cell group in spleen (left) and thymus (right). Splenocytes and thymocytes harvested from mice injected PBS, PD-L1-Fc (0.8 mg/kg), sPD-L1 mRNA-MC3-LNPs (0.2 mg/kg) or sPD-L1 mRNA-DOTAP-LNPs (0.2 mg/kg). Flow cytometric analysis of CD4+ T cells (CD4+ T), CD8+ T cells (CD8+ T), CD4+ CD25+ regulatory T cells (Tregs), dendritic cells (DCs) and macrophages (MΦs) from each group at different ratios. *p < 0.05, **p < 0.01. t-test analysis. (L) Seven-day survival rate of mice treated with different agents, including sham, PBS, PD-L1-Fc (0.8 mg/kg), sPD-L1 mRNA-MC3-LNPs (0.2 mg/kg), or sPD-L1 mRNA-DOTAP-LNPs (0.2 mg/kg) (n = 15). **p < 0.01 by log-rank test.

In subsequent experiments, we investigated the efficacy of lung-targeted delivery of sPD-L1 mRNA using mRNA-DOTAP-LNPs in attenuating acute immune response. ARDS mice were intravenously administered with the same dosage (0.2 mg/kg) of either sPD-L1 mRNA-MC3-LNPs or mRNA-DOTAP-LNPs. We measured various inflammatory indicators, including WBC counts, protein concentration in BALF, wet/dry weight ratio, and levels of inflammatory cytokines (Figure 6A-D). Additionally, we evaluated the extent of lung injury observed in pathological sections (Figure 6E, Figure S5A). Notably, we observed that both types of sPD-L1 mRNA-LNPs demonstrated comparable reductions in the inflammatory response across all these indicators.

Next, we evaluated the therapeutic effect of sPD-L1 mRNA-DOTAP-LNPs (0.2 mg/kg) on ARDS, comparing it with PBS, PD-L1-Fc recombinant protein (0.8 mg/kg), and Luc mRNA-DOTAP-LNPs (0.2 mg/kg). In comparison to all these controls, the administration of sPD-L1 mRNA-DOTAP-LNPs demonstrated significant reductions in protein accumulation, WBC recruitment, edema, cytokine levels, and lung pathology (Figure 6F-J, Figure S5B). Based on the above findings, a single dose of sPD-L1 mRNA-DOTAP-LNPs led to an elevation in expression of sPD-L1 over 3 days post injection, effectively reducing the acute immune response.

To confirm the specific immunosuppressive effects of sPD-L1 mRNA-DOTAP-LNPs in lung tissue, we conducted a comprehensive examination to validate the specific immunosuppressive effects of sPD-L1 mRNA-DOTAP-LNPs in the lungs while minimizing the impact on immune cells in other tissues. We analyzed changes in immune cell populations in the thymus and spleen of mice injected with a dosage of 0.2 mg/kg sPD-L1 mRNA-DOTAP-LNPs. Compared to the control group, the intervention with sPD-L1 mRNA-DOTAP-LNPs did not significantly affect the proportions of immune cells in the spleen and thymus. However, after the administration of 0.8 mg/kg PD-L1-Fc, dendritic cells in the spleen increased by approximately 2-fold, and after the injection of 0.2 mg/kg sPD-L1 mRNA-MC3-LNPs, a substantial number of macrophages were detected in the spleen. This result suggests that localized immune suppression by sPD-L1 mRNA-DOTAP-LNPs can reduce the impact on immune cells in other tissues in comparison to such effects of PD-L1-Fc protein and sPD-L1 mRNA-MC3-LNPs administration (Figure 6K, Figure S6).

To confirm the impact of sPD-L1 intervention on the survival period of ARDS-modeled mice, we monitored the effect of a single dose of PD-L1-Fc (0.8 mg/kg), sPD-L1 mRNA-MC3-LNPs (0.2 mg/kg), and sPD-L1 mRNA-DOTAP-LNPs (0.2 mg/kg) on the 7-day survival rates of the ARDS mice (Figure 6L). The PBS intervention group showed rapid mortality within 3 days, with only 30% of the mice surviving. In contrast, the administration of a single dose of sPD-L1 mRNA-DOTAP-LNPs significantly extended mouse survival rates, achieving a survival rate of 87% after 7 days. Meanwhile, mice treated with PD-L1-Fc and sPD-L1 mRNA-MC3-LNPs exhibited similar survival rates, with 60% and 67%, respectively (Figure 6L). These compelling results provide strong support for the notion that *in situ* pulmonary expression of sPD-L1 can effectively protect mice from death induced by acute inflammation.

Furthermore, we assessed the* in vivo* safety profile of mRNA-DOTAP-LNPs by measuring changes in lung tissue bacterial colony-forming units (CFU) compare to the PBS-treated group in PAO-induced mice after administering sPD-L1 mRNA-DOTAP-LNPs (0.2 mg/kg). Notably, our findings indicated that the *in-situ* expression of sPD-L1 did not hinder bacterial clearance in lung tissue (Figure S7A). To assess potential immune responses caused by DOTAP-LNPs, we measured the concentrations of TNF-α and IL-6 in the BALF after injection of sPD-L1 mRNA-DOTAP-LNPs (0.2 mg/kg), as well as conducted histopathological analysis of vital organs. Our results revealed no significant immune response, as evidenced by the negligible changes in TNF-α and IL-6 levels, along with the absence of histopathological abnormalities in vital organs (Figure S7A-C). Furthermore, DOTAP-LNPs have been reported not to cause any adverse effects on lung, liver, and spleen cells 41. These comprehensive findings collectively demonstrated the favorable safety profile of mRNA-DOTAP-LNPs, affirming their potential as a safe and effective therapeutic option for the treatment of acute inflammatory conditions.

## Discussion

In this study, we demonstrated that soluble programmed death ligand-1 (sPD-L1) mRNA delivered using DOTAP-added lipid nanoparticles (LNPs) can be expressed by lung tissue specifically, achieving *in situ* immune suppression in ARDS lung tissue. This approach effectively attenuated acute immune response and prolonged the seven-day survival rate of mice with acute lung injury, providing a proof-of-concept for the use of mRNA therapy in acute inflammatory disease.

In our study, we opted to express soluble PD-L1 (sPD-L1) to inhibit excessive immune responses, thereby highlighting the robust immunoregulatory potential of sPD-L1. This soluble form retains the extracellular domain of PD-L1 while lacking the transmembrane and intracellular domains. Here we reported that sPD-L1 can ameliorate immune cell accumulation, decrease inflammatory cytokine secretion, and even mitigate tissue swelling, indicating its potential as a therapeutic target. Furthermore, in a previous observational clinical study, we observed that low levels of serum sPD-L1 were associated with more severe disease and predicted a worse prognosis in patients with ARDS 19. These collective findings strongly support the notion that sPD-L1 possesses considerable potential as both a diagnostic biomarker and a target for therapeutic interventions.

In addition to sPD-L1, numerous other immune inhibitory factors have been documented 42. These findings imply that alternative cancer targets, such as the B7-CD28 superfamily proteins, may harbor significant therapeutic prospects in immune-related disorders. Notably, PD-L1 can engage with both PD-1 and B7-1 surface proteins, endowing it with a potential advantage over other immune inhibitory molecules like CTLA-4 43.

Protein therapy has emerged as a valuable approach in the treatment of various diseases, with more than 100 protein and peptide-based therapeutic drugs currently approved for clinical use. However, the production of therapeutic proteins typically involves costly chemical synthesis or recombinant cell production methods. Fluctuations in external or internal factors such as temperature, pH, and chemical environment can lead to protein denaturation, aggregation, and precipitation, which may trigger immune responses in patients 44. In contrast, mRNA therapy harnesses the cell's own ribosomes to translate therapeutic proteins, enabling proper protein folding and post-translational modifications. This circumvents the issues associated with storage conditions and high immunogenicity. To highlight the advantages of mRNA therapy, we compared the *in vivo* half-life and therapeutic effects of sPD-L1 mRNA-LNPs with a commercially available PD-L1-Fc recombinant protein in a murine model of ARDS. Our findings revealed that the mRNA-LNPs strategy exhibited superior pharmacokinetic characteristics and demonstrated a comparable half-life to PD-L1-Fc at lower doses. Importantly, it should be noted that while both protein therapy and mRNA-LNPs demonstrated therapeutic efficacy, the treatment with sPD-L1 mRNA-DOTAP-LNPs showed superior effectiveness in prolonging the survival of ARDS mice. These findings highlight the potential of mRNA-based therapy as a promising approach for the treatment of ARDS, offering a more robust and durable therapeutic effect compared to traditional protein-based therapies. Further research is warranted to explore and optimize the therapeutic potential of mRNA-based therapies in diverse disease contexts.

Western blot assay was performed to confirm the protein expression of the modified mRNA and we detected a 40 kDa precursor protein in the whole cell lysate, and a 50 kDa protein in the supernatant. Previous reports showed that the non-glycosylated form of PD-L1 is about 33 kDa, while the immunoblot band is 50 kDa after glycosylation is complete 45. This indicates that the sPD-L1 protein translated from the IVT mRNA template secrets normally and undergoes glycosylation. This ability to utilize the cell's intrinsic post-translational modification system for protein modification represents a distinctive advantage of mRNA therapy that cannot be replicated by protein therapy. In addition, the detection of protein expression in the supernatant indicates that sPD-L1 can be secreted normally.

The development of tissue-specific delivery systems, like SORT LNPs, holds great promise for achieving localized immune suppression, avoiding disruptions to global immune balance while reducing excessive inflammatory responses. In this study, both systemic and lung tissue-specific immunosuppression have demonstrated promising inhibitory effects in treating ARDS, yet they entail distinct therapeutic strategies and considerations. Upon administering PD-L1-Fc and sPD-L1 mRNA-MC3-LNPs to mice, we observed changes in the proportions of immune cell subpopulations in the spleen. PD-L1-Fc led to an increase in regulatory T cells, while sPD-L1 mRNA-MC3-LNPs resulted in an elevated population of macrophages. Notably, lung-targeted sPD-L1 mRNA-DOTAP-LNPs administration successfully avoided these undesired changes in immune cell subpopulations. This feature underscores a key advantage of local immune suppression: its ability to preserve overall immune function. In contrast, systemic immune suppression often leads to a broad inhibition of immune system functions, including defense against pathogens and clearance of tumors. Our findings highlight the importance of optimizing delivery strategies to maximize the therapeutic potential of tissue-specific immune modulation.

In addition to our exploration of lung tissue-specific delivery achieved through SORT LNPs, we also tested aerosol inhalation administration. However, we observed approximately 100-fold lower expression levels in lung tissue after 6 hours when compared to the intravenous administration of DOTAP-LNPs. Furthermore, the aerosolization process resulted in a 30% loss of LNPs. These limitations prompted us to refrain from further assessing the feasibility of aerosol inhalation as a potential therapeutic approach. The nebulizer we utilized is designed with an extended catheter inserted into the trachea of mice, thereby facilitating the immediate administration of the aerosolized solution and efficaciously delivering nebulized droplets directly to the lungs. This method indeed differentiates from the conventional human aerosol inhalation approach. We hold the perspective that the potential adoption of a direct nebulizer equipped with a nose mask to replicate human inhalation patterns could potentially offer a more advantageous method for aerosol administration. However, we acknowledge that this approach necessitates rigorous further evaluation and consideration in the times ahead.

We observed that sPD-L1 mRNA-LNPs demonstrated therapeutic effects on protein concentration, leukocyte count, and lung edema in BALF, consistent with our previous research findings. In our previous study, sPD-L1 was shown to induce apoptosis in monocyte-derived macrophages (MDMs) 19. This could potentially contribute to decrease the release of inflammatory and chemotactic factors. As a result, it may mitigate immune cell infiltration and minimize the damage caused by excessive immune responses, such as protein accumulation and tissue edema. Interestingly, even the Luc mRNA-LNPs group exhibited a certain degree of immunosuppressive effect compared to the PBS control group. It has been reported that approximately 3% of LNPs are internalized by the circulating leukocytes following intravenous administration 46. We speculate that the internalization of LNPs by immune cells may trigger apoptosis or elicit immune suppression-related responses. However, there is currently limited information regarding the immunosuppressive effects of LNPs on immune cells, and further investigations are needed to elucidate this mechanism.

Certainly, there is still significant work ahead before clinical translation can be achieved. Although we conducted histopathological analysis to assess the safety of sPD-L1 mRNA-DOTAP-LNPs in lungs and other vital tissues of mice, the safety of the drugs needs further assessed. Potential questions may include cell membrane damage caused by the cationic lipid DOTAP present in the LNP formulation and potential inhibition of the lung tissue's antibacterial properties due to sPD-L1 expression. To address the latter concern, exploring whether combination therapies involving antibiotics or antiviral agents could enhance the therapeutic efficacy is necessary. In fact, ARDS patients may receive various medications during the treatment process, including antibiotics for the prevention or treatment of potential bacterial infections, diuretics, vasopressors, and corticosteroids to maintain hemodynamic stability and fluid balance 1, 47, 48. Bronchodilators may also be administered. sPD-L1 mRNA-DOTAP-LNPs can be used as an immunomodulator in combination with these other medications to achieve the best therapeutic outcomes. Therefore, further comprehensive experimental evaluations are warranted to investigate these potential applications. Furthermore, although administering equivalent doses of sPD-L1 mRNA-MC3-LNPs and mRNA-DOTAP-LNPs resulted in comparable treatment outcomes, significant variations were observed in the expression levels of sPD-L1 within the lung tissue, highlighting the need for further evaluation to determine the optimal dosage for effective patient treatment.

In conclusion, the significant potential of mRNA therapy has been demonstrated in this study, providing valuable insights into the effective application of tissue-specific targeted SORT LNPs-mediated mRNA therapy for localized immune suppression, opening up new avenues for the development of improved treatment strategies for ARDS and potentially other immune-related diseases.

## Supplementary Material

Supplementary figures.

## Figures and Tables

**Table 1:** Lung injury scoring scale

Parameter	Score per field		
	0	1	2
A. Neutrophils in the alveolar space	none	1 - 5	> 5
B. Neutrophils in the interstitial space	none	1 - 5	> 5
C. Hyaline membranes	none	1	> 1
D. Proteinaceous debris filling the airspaces	none	1	> 1
E. Alveolar septal thickening	< 2×	2× - 4×	> 4×

Score = [(20 × A) + (14 × B) + (7 × C) + (7 × D) + (2 × E)]/100

## Abbreviations

ARDS: acute respiratory distress syndrome
LNPs: lipid nanoparticles
sPD-L1: soluble programmed death ligand-1
mRNA: messenger RNA
BALF: bronchoalveolar lavage fluid
MDMs: monocyte-derived macrophages
IVT: *in vitro* transcribed
SORT: selective organ targeting
TEM: transmission electron microscopy
HE: hematoxylin and eosin
unmod: unmodified
mo^5^U: 5-methoxyuridine
m^5^C/ψ: 5-methylcytosine and pseudouridine
ψ: pseudouridine
m^1^ψ: N_1_-methylpseudouridine
PDI: polydispersity index
Luc: luciferase
ICs: immune cells
MΦs: Macrophages
ECs: Endothelial cells
EpiCs: epithelial cells
Fc: fragment crystallizable region
IgG1: immunoglobulin G1
PAO: *Pseudomonas aeruginosa*
WBC: white blood cell
