# Supplementary material for: Application of Lung-Targeted Lipid Nanoparticle-delivered mRNA of soluble PD-L1 via SORT Technology in Acute Respiratory Distress Syndrome
Source: Theranostics. 2023 Sep 4;13(14):4974–92. doi: 10.7150/thno.86466 (PMC10526659; doi:10.7150/thno.86466)
Supplement: Supplementary file 1 — Supplementary figures. [file thnov13p4974s1.pdf]

Supplementary figures:

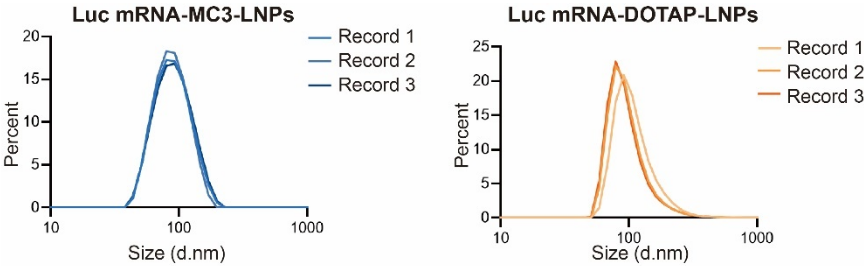

Figure S1. Particle size distribution of Luc mRNA-LNPs.

Particle size distribution of Luc mRNA-MC3-LNPs and mRNA-DOTAP-LNPs. Each data was recorded 3 times. d.nm, diameter (nm).

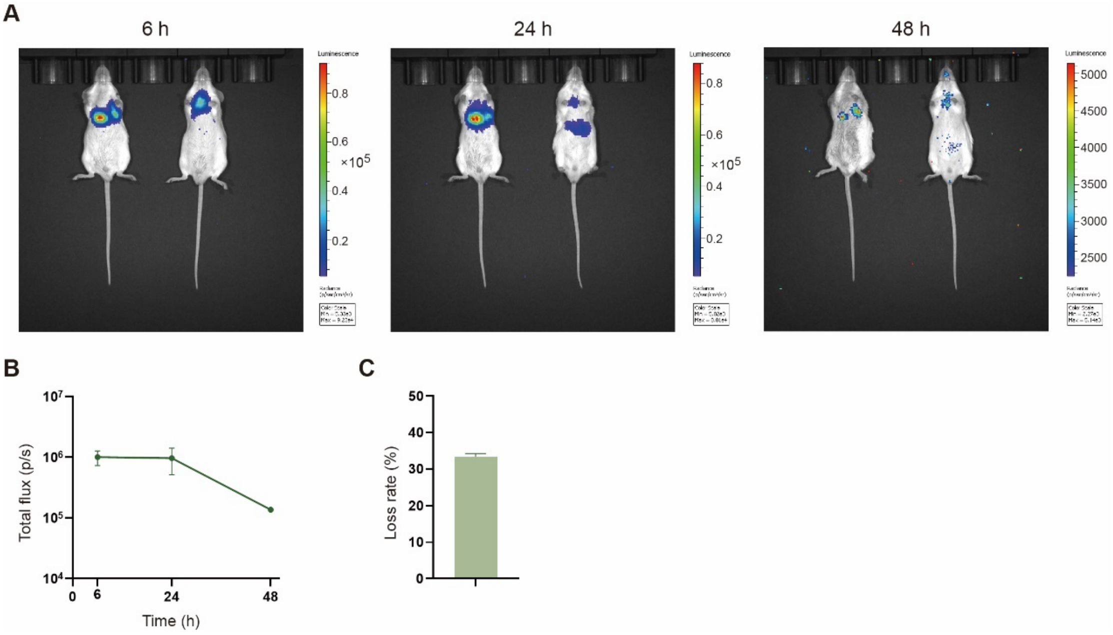

Figure S2. Mice were administered aerosolized mRNA-MC3-LNPs to the lungs via nebulization.

(A) Representative whole-body bioluminescence images of mice at various time points (6 h, 24 h, 48 h) after administration of 0.4 mg/kg Luc mRNA-MC3-LNPs via nebulization. Luminescence scale varies between time points and groups. (B) Quantification of bioluminescence intensity of (A). Results represent mean ± SEM (n = 2). (C) Percentage loss of mRNA-LNP after nebulization. Results represent mean ± SEM (n = 3).

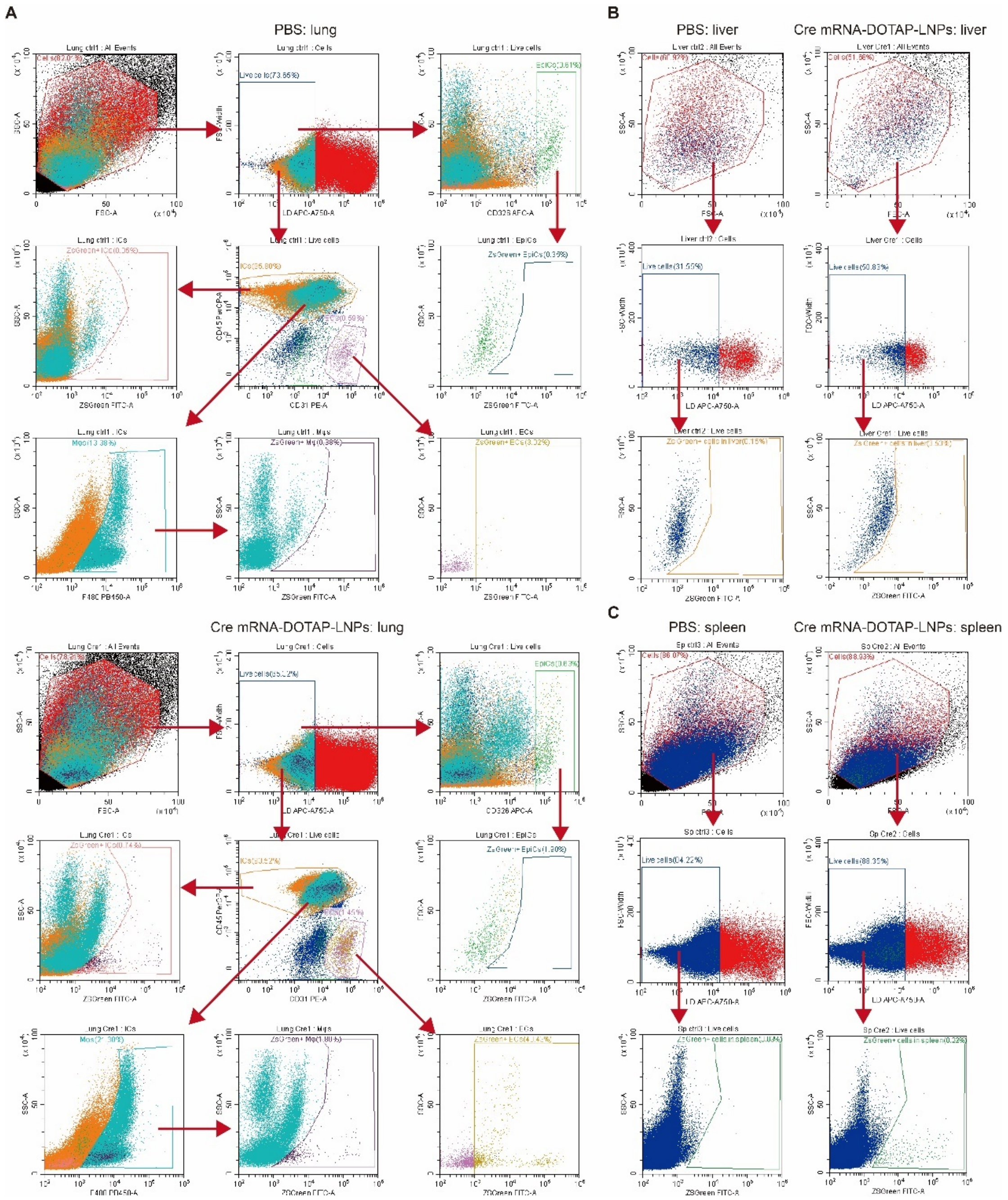

Figure S3. Flow cytometry gating strategy for ZsGreen+ expression analysis in lung, liver and spleen cells.

Mice in Cre group received an injection of 0.8 mg/kg Cre mRNA-DOTAP-LNPs while the control (ctrl) group was injected with PBS. After two days, cells from the lung (A), liver (B) and spleen (C) were collected for FACs analysis. FVS780 was used to distinguish live and dead cells. Immune cells (ICs) were identified using PerCP/Cy5.5-CD45 antibody. Macrophages (MΦs) were labeled with BV421-F4/80 antibody. Endothelial cells (ECs) were marked with PE-CD31 antibody and epithelial cells (EpiCs) were stained with APC-CD326 antibody (n = 3).

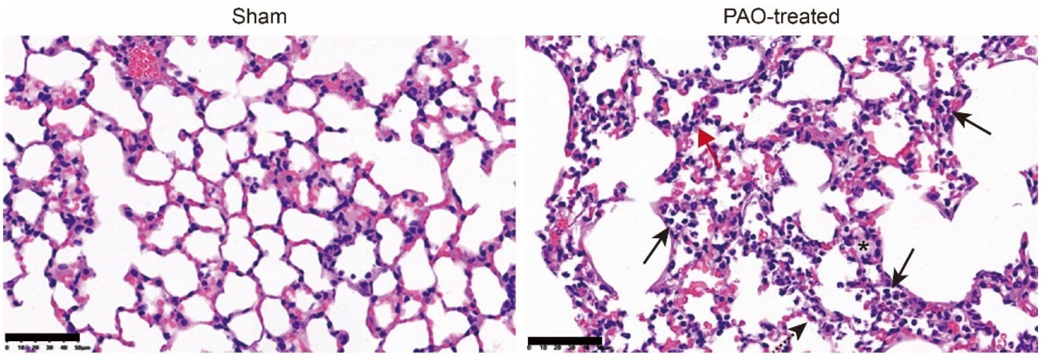

Figure S4. Representative histological images for lung injury scoring.

The sham group (left, score = 0) exhibited thin alveolar septa and mostly cell-free airspaces. In the PAO-treated group (right, score = 0.77), black arrows indicate alveoli with >5 neutrophils, score = 2. Red arrows indicate interstitial spaces with >5 neutrophils, score = 2. An asterisk denotes proteinaceous debris filling one alveolar space, score = 1. Alveolar septal thickening is almost 2 times greater, score = 1. Total score = [(20×2) +(14×2) + (7×0) + (7×1) +(2×1)]/100 = 0.77. Scale bar, 50 μm.

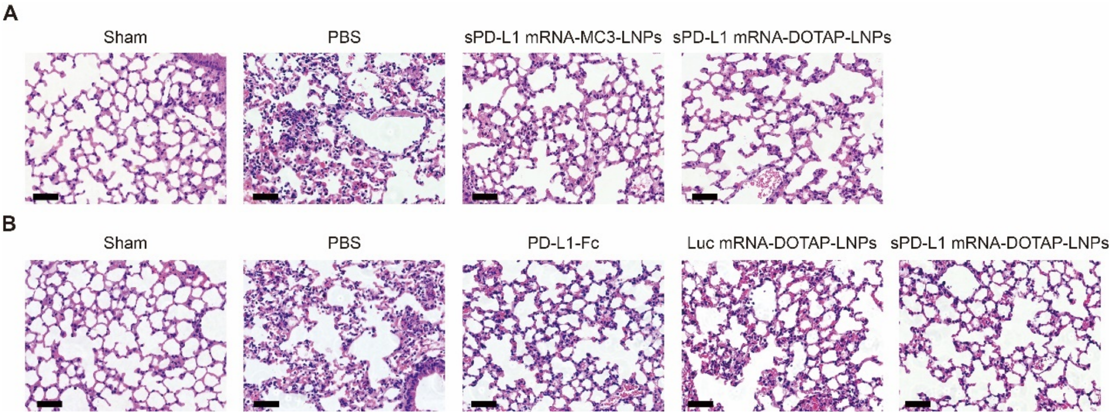

Figure S5. Representative histological images for lung injury scoring in different drug-treated groups.

(A) Representative images of HE-stained sections from Figure 6E, showing the following treatment groups: sham, PBS, sPD-L1 mRNA-MC3-LNPs (0.2 mg/kg), and sPD-L1 mRNA-DOTAP-LNPs (0.2 mg/kg). Scale bar, 50 μm. (B) Representative images of HE-stained sections from Figure 6J, illustrating the following treatment groups: sham, PBS, PD-L1-Fc (0.8 mg/kg), Luc mRNA-DOTAP-LNPs (0.2 mg/kg), and sPD-L1 mRNA-DOTAP-LNPs (0.2 mg/kg). Scale bar, 50 μm.

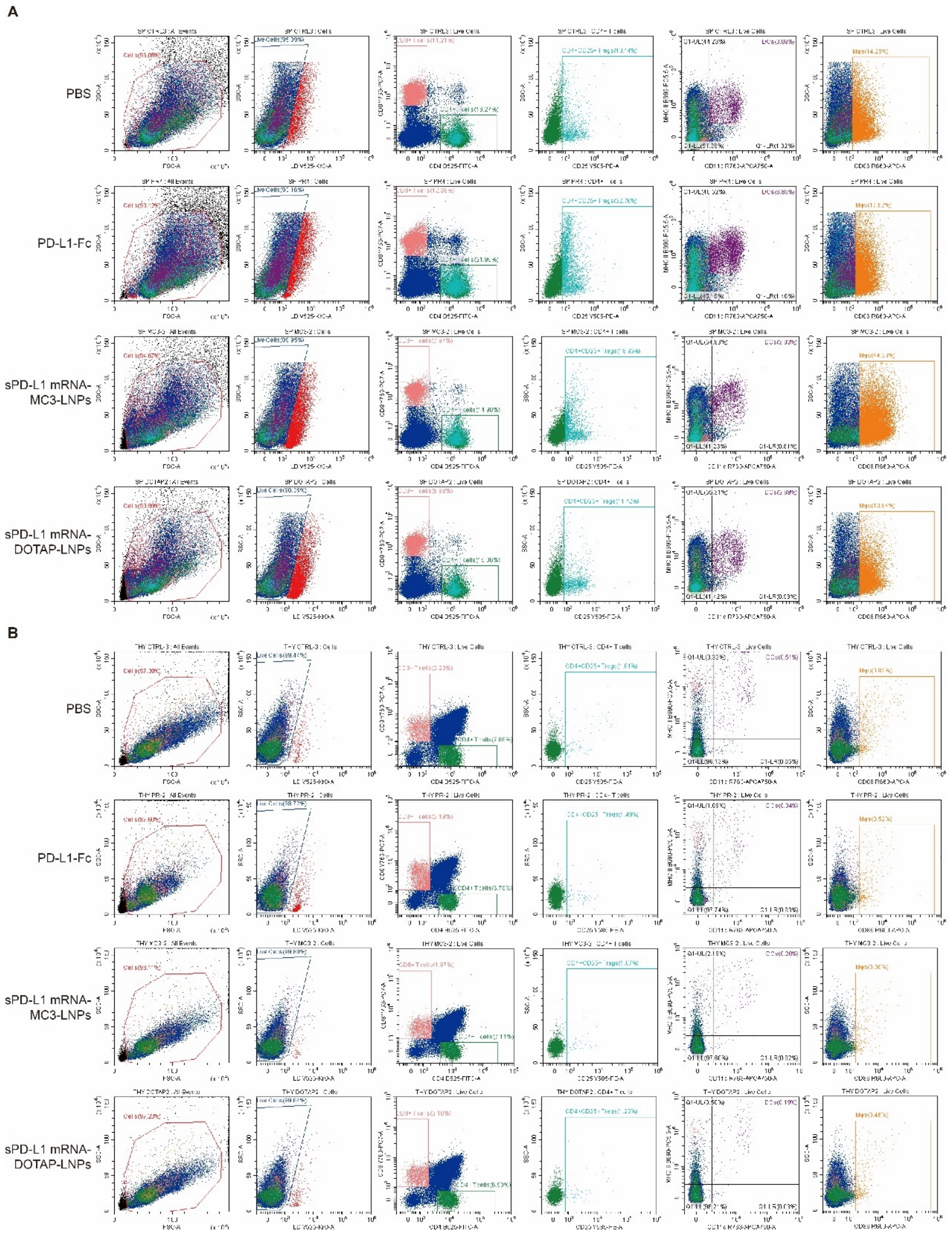

Figure S6. Flow cytometry gating strategy for cell populations in the spleen and thymus.

Three days after injection of PBS, PD-L1-Fc (0.8 mg/kg), sPD-L1 mRNA-MC3-LNPs (0.2 mg/kg) or sPD-L1 mRNA-DOTAP-LNPs (0.2 mg/kg), mouse splenic (A) and thymic cells (B) were harvested. FVS510 was used as a viability dye to discriminate between live and dead cells. CD4+ T cells were gated using FITC-CD4+ antibody, while CD8+ T cells were gated using PE/Cy7-CD8+ antibody. Within the CD4+ T cell population, Treg cells were gated using PE-CD25 antibody. Dendritic cells (DCs) were gated using PerCp/Cy5.5-MHCII and APC/Cy7-CD11c antibodies, and macrophages (MΦs) were gated using APC-

CD86 antibody (n = 3).

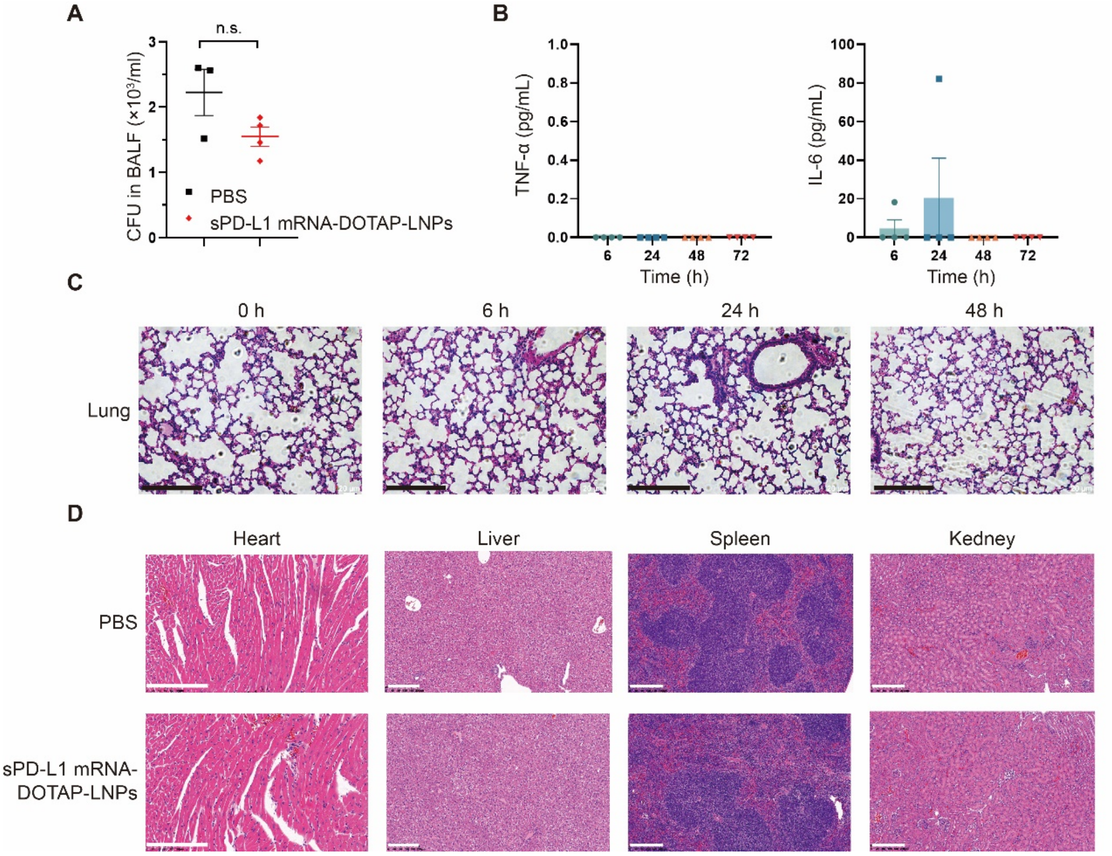

Figure S7. Safety profile of sPD-L1 mRNA-DOTAP-LNPs.

(A) Detection of the impact of sPD-L1 mRNA-DOTAP-LNPs on bacterial clearance in the lung. 4 hours after PAO modeling, PBS or sPD-L1 mRNA-DOTAP-LNPs (0.2 mg/kg) were administered via tail vein injection. After 12 hours, bronchoalveolar lavage fluid (BALF) was collected from mice and plated on agar plates. The plates were then placed in a 37 °C bacterial incubator for one day to assess bacterial colony counts. Results represent mean ± SEM (n⩾3). (B) Concentration of inflammatory factor (TNF-α, IL-6) in the BALF at different time points (0 h, 6 h, 24 h, 48 h) after intravenous administration of sPD-L1 mRNA-DOTAP-LNPs (0.2 mg/kg). Results represent mean ± SEM (n = 4). (C) Histopathological analysis of lung tissues at different time points (0 h, 6 h, 24 h, 48 h) after intravenous administration of sPD-L1 mRNA-DOTAP-LNPs (0.2 mg/kg). Scale bar, 100 μm. (D) HE staining was performed on heart, liver, spleen, and kidney sections obtained from the PBS control group and the sPD-L1 mRNA-DOTAP-LNPs treatment group (0.2 mg/kg) at the 12-hour time point. Scale bar, 200 μm.
